# Storage Phosphors for Medical Imaging

**DOI:** 10.3390/ma4061034

**Published:** 2011-06-09

**Authors:** Paul Leblans, Dirk Vandenbroucke, Peter Willems

**Affiliations:** 1 Agfa HealthCare NV, Septestraat 27, B-2640 Mortsel, Belgium; E-Mail: dirk.vandenbroucke@agfa.com; 2 Industrial Scientific and Computing BVBA, Hellestraat 55, B-9190 Stekene, Belgium; E-Mail: peter.willems.pw@gmail.com

**Keywords:** computed radiography, storage phosphor, X-ray, optically stimulated lumi-nescence, BaFBr:Eu^2+^, CsBr:Eu^2+^, needle imaging plate, image quality

## Abstract

Computed radiography (CR) uses storage phosphor imaging plates for digital imaging. Absorbed X-ray energy is stored in crystal defects. In read-out the energy is set free as blue photons upon optical stimulation. In the 35 years of CR history, several storage phosphor families were investigated and developed. An explanation is given as to why some materials made it to the commercial stage, while others did not. The photo stimulated luminescence mechanism of the current commercial storage phosphors, BaFBr:Eu^2+^ and CsBr:Eu^2+^ is discussed. The relation between storage phosphor plate physical characteristics and image quality is explained. It is demonstrated that the morphology of the phosphor crystals in the CR imaging plate has a very significant impact on its performance.

## 1. Introduction

### 1.1. Medical Radiography

Almost immediately following the discovery by W.C. Röntgen in 1895, X-rays have been in use in medical imaging. In medical radiography, shadow images are made of the internal structures of the human body by placing the patient between an X-ray source and an X-ray image detector. Medical X-ray spectra have quanta with energies between 10 and 150 keV, depending on the application. X-ray imaging is based on the fact that the quanta are very penetrative and that different materials have large differences in intrinsic absorption. In the medical energy range the X-ray absorption cross-section of materials is proportional to their density and to the 4th power of the atomic number of their elements. Bones, having a large Ca content, have a much higher intrinsic absorption than soft tissue.

Another consequence of the very high penetration power is that X-ray film with its about 5 μ thick active silver halide (AgX) layer has a very low X-ray sensitivity. A very small fraction of the quanta interacts with the AgX crystals of the film emulsion. Only in non-destructive testing (NDT) are X-ray images made directly on film. The doses needed to make a useful image directly on film are much too high for medical imaging.

### 1.2. Screen/Film Radiography

**Figure 1:**
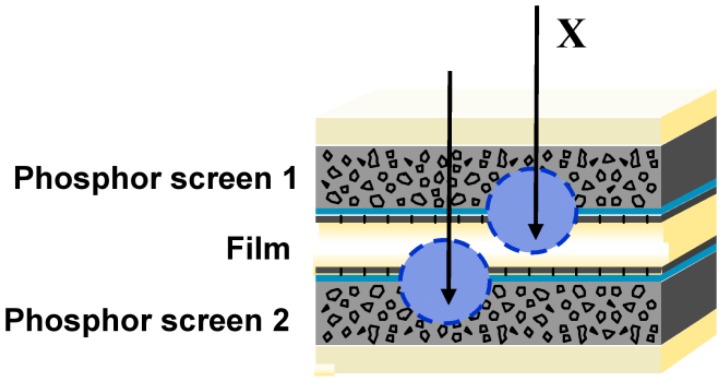
Screen / film detector, used in conventional radiography.

In screen/film radiography, therefore, the AgX film is sandwidged between 2 phosphor screens (Figure 1).

X-ray phosphors are inorganic luminescent crystalline powders that convert X-rays into visible light. Visible photons are generated in three steps. X-ray energy is absorbed and dissipated into the material. Depending on the X-ray quantum energy, dissipation may result from the photoelectric effect, from Compton scattering or from pair production. In each case the energy is finally converted into phonons and in mobile electrons and holes. When these free charge carriers have given up enough energy to the lattice they can combine to form excitons. The excitons transfer their energy to a luminescent center, where it is converted into a photon.

In order to have a high X-ray interaction cross-section, X-ray phosphors contain high atomic number elements and have densities of 5 g/cm^3^ or more. The phosphor transforms the X-ray image into a light image, which creates a latent image in the photographic film. The first step in making the X-ray image is to expose the screen/film sandwich in a light-tight cassette to the X-ray beam that has passed through the patient. After X-ray exposure, the photographic film is taken out of the cassette, introduced automatically into a development machine and developed into a so-called hard-copy image.

### 1.3. Computed Radiography (CR)

About 20 years ago, X-ray radiography was the only non-digital medical imaging technique. Technically, fluoroscopy was not digital either, but since it produces moving images it is considered separate. X-ray images had to be stored in voluminous file cabinets and separate, inefficient image retrieval procedures were needed. Image exchange was impractical. Apart from efficient storage and retrieval, advantages of digital imaging are the possibility of image processing for detail visibility improvement. Further, thanks to the fact that digital detectors have a much wider dynamic range than film, under- or over-exposure is avoided. This eliminates the need for retakes that occurs with screen/film systems [1].

The search for digital radiographic technologies started in the seventies of last century. Kodak had the luck to patent the principle of computed radiography (CR) in the middle of this decade [2]. CR is often compared to direct radiography (DR). Where CR generally involves the use of a cassette, like in traditional screen/film systems, DR typically captures the image directly onto a flat panel detector. Most DR detectors have a phosphor screen to convert the X-ray image into a visible light image. The light creates a digital output signal in an a-Si photodiode layer. The digital signal is then read out by thin film transistors.

**Figure 2:**
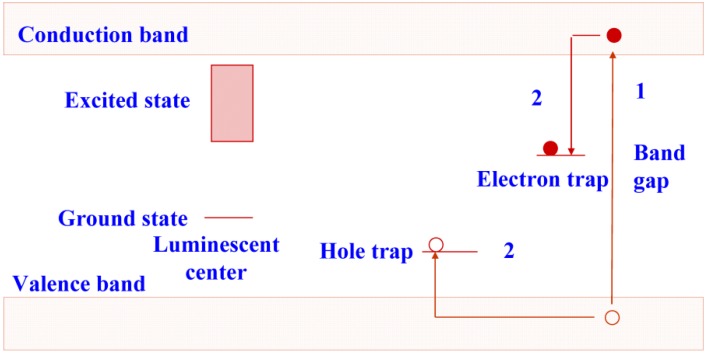
Energy diagram showing electron and hole trapping in a storage phosphor.

In CR, the conventional X-ray phosphor screen + film detector is replaced by a storage phosphor plate. Evidently, the storage phosphor is responsible for substantial X-ray absorption. Unlike what happens in conventional phosphors, in a storage phosphor, part of the electron/hole pairs do not recombine to transfer their energy to a luminescent center. A considerable fraction is trapped in metastable states. A simplified non-detailed general model of the storage process is shown in Figure 2. As long as the phosphor is not exposed to light or heat, recombination is not possible and the electrons and holes remain trapped. The spatial distribution of the trapped charges in a plate containing storage phosphor crystals makes up the latent image in CR.

**Figure 3:**
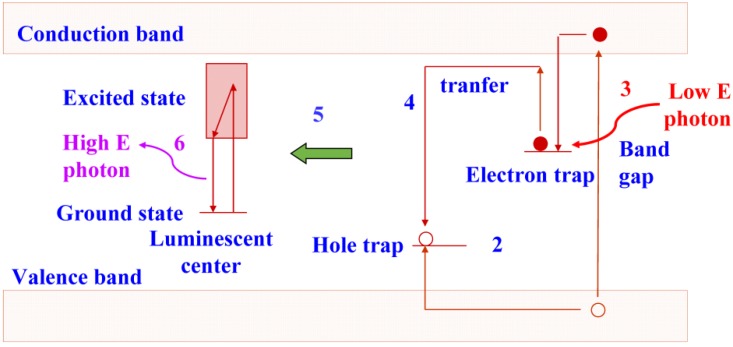
Energy diagram showing photo-stimulated luminescence in a storage phosphor.

The trapped electrons are sensitive to light. In an efficient storage phosphor, a red or near-infrared photon supplies sufficient energy to escape. Subsequently, recombination with a trapped hole is possible, followed by energy release initiating luminescence as in a conventional phosphor (Figure 3). The photons emitted by the storage phosphor have higher energy than the photons stimulating the trapped electrons, *i.e.*, the process is anti-Stokes. It is called photo-stimulated luminescence (PSL) in contrast to the conventional luminescence, where a lower energy photon is generated by a higher energy photon and which is called excited luminescence.

**Figure 4:**
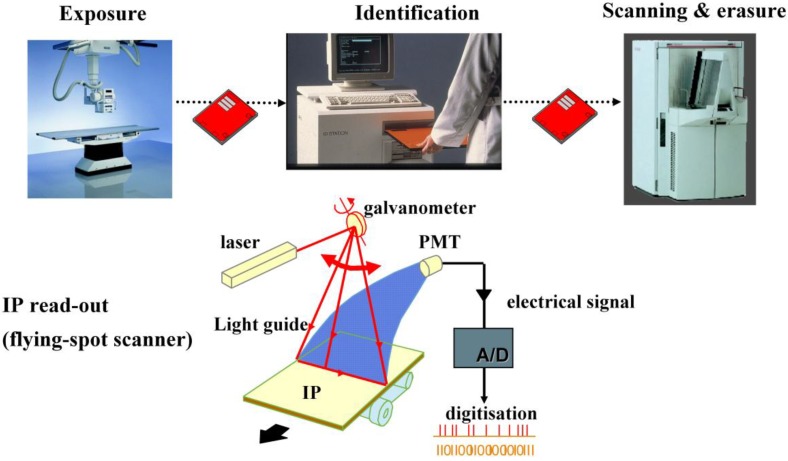
CR storage phosphor plate exposure and read-out.

In the CR work-flow a storage phosphor plate is exposed in a light-tight cassette as in screen/film radiography. After X-ray exposure and patient identification, the cassette is introduced in a scanner for read-out (Figure 4).

The imaging plate (IP) is taken out of the cassette for scanning. A red laser beam is moved over the plate in the “fast-scan direction” by a scanning galvanometer or rotating mirror. Simultaneously, the plate moves slowly in the “slow-scan direction”. In this way, the pixels of the storage phosphor plate are stimulated one by one. The local storage phosphor emission is proportional to the local X-ray absorption in the plate. A light guide transfers the emitted light to a photomultiplier tube (PMT). A filter in front of the PMT absorbs the reflected laser light and transmits the phosphor emission light (Figure 4). In the PMT, the light signal is transformed into an electrical signal, amplified, digitized and stored in a computer file. After erasure the plate is re-inserted into the cassette for re-use.

## 2. CR Phosphor Requirements

A good storage phosphor for medical CR must meet the following requirements [3]:

1. It should have a high X-ray absorption for medical X-ray spectra, *i.e*., for energies ranging from 20 keV to 140 keV.
2. It should have high conversion efficiency. The amount of energy stored per unit X-ray energy should be high. This implies that a large fraction of the mobile charge carriers created by the absorbed X-ray quanta is converted into trapped electrons and holes forming defect aggregates leading to PSL. The energy needed to create a trapped electron and hole in the storage phosphors in commercial use is estimated to be:
  100 eV in BaFBr:Eu^2+^
  67 eV in CsBr:Eu^2+^.
  This means that one absorbed 50 keV X-ray quantum (general radiography) creates 500 to 750 trapped charge pairs an one absorbed 20 keV quantum (mammography) 200 to 300.
3. The image information stored in the phosphor should have slow fading in dark at room temperature, *i.e*., electron- and hole traps should be stable. In practice, the so-called dark-decay of the stored image in a CR plate is between 10 and 25% in the first hour after X-ray exposure.
4. The stimulation spectrum should be in the range covered by inexpensive solid state lasers. Currently laser diodes are commercially available with wavelengths ranging from 375 nm to 1800 nm, but red and IR lasers exhibit the best price/performance ratio. In addition, the stimulation light should not produce free charges that can give rise to PSL. This excludes lasers with wavelengths in the blue and UV parts of the spectrum. Finally, the stimulation light and the stimulated emission should be spectrally separable. This is necessary since the emitted light is between 10^5^ and 10^9^ times weaker than the stimulation light, depending on X-ray spectrum and dose used to make the image. Color filters are applied to block the stimulation wavelength and transmit the emission light. Since the slope of the filter curves is finite, a stimulated emission with a wavelength close to that of the laser will also be attenuated. Altogether, this means that efficient stimulation of the trapped charges should be possible in the wavelength range between 500 and 1,500 nm. However, it is unlikely that trapped charges that can be stimulated with 1,000 to 1,500 nm light are stable at room temperature in the dark.
5. The CR phosphor emission should match the sensitivity spectrum of the light detector (Figure 5). In flying-spot scanners with a PMT light detector, the emission is preferable below 500 nm and most preferably in the range between 350 and 450 nm.

**Figure 5:**
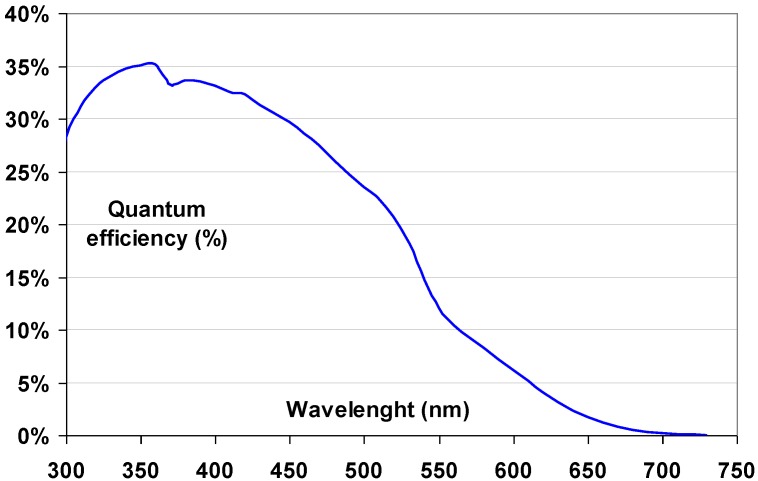
Quantum efficiency of a typical CR scanner PMT.
6. The laser power required for CR plate stimulation should be low. This implies that both the oscillator strength and the recombination probability of the stimulated trap should be high. Actual CR scanner laser powers are between 5 and 30 mW.
7. Since read-out of a complete image plate in less than one minute is desirable, the luminescence decay time should be short. In current flying-spot CR digitizers, the pixel read-out time does not exceed 2 µs. At the time the next pixel is stimulated, the light emission of the previous pixel should have decayed to at least 1/e of its initial value. Consequently, for use in flying-spot scanners the decay time should be maximum 2 µs.
  For line scanners phosphor decay time is much less critical. In these devices a complete line is stimulated and read-out at once and the line-time is typically in the millisecond range.
8. The IP should be re-usable. Only roughly 50% of the stored energy (trapped charges) is released during scanning. Hence, the IP must be erased for re-use. For erasure the phosphor is illuminated with a strong light source. To allow efficient erasure, the amount of “persistent traps” that is formed upon X-ray radiation should be negligible.
9. The phosphor should be stable under normal room conditions, *i.e.*, its performance should not degrade when it is exposed to humidity and daylight.
10. It should be possible to manufacture the phosphor on an industrial scale in a commercially viable way.
11. Spatial and contrast resolution are important clinical requirements and are affected by phosphor quality as well. Smaller particles lead to higher resolution and to lower light output. The best compromise is obtained when the phosphor particles have a median size in the range between 1 and 10 μ.

## 3. Powder Imaging Plates (PIP’s) for Medical CR

### 3.1. PIP Manufacturing

**Figure 6:**
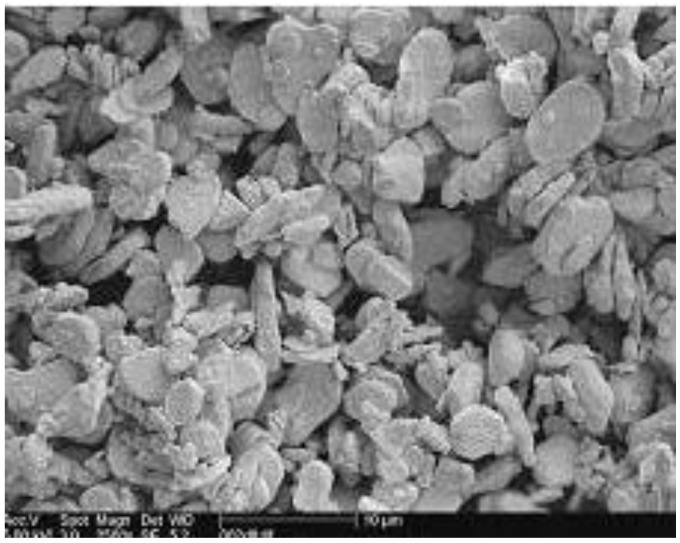
SEM image of BaFBr:Eu^2+^ storage phosphor powder.

Traditional X-ray phosphor plates are powder plates, based on phosphor crystals with a median particle size of 2 to 15 μ (Figure 6). Individual particles may be sub-μ or as large as 30 to 40 μ.

**Figure 7:**
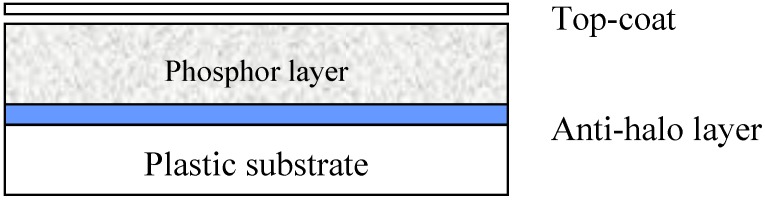
Drawing of typical PIP cross-section.

Phosphors are crystalline host materials, almost always containing a trace amount of an activator element. The activator molecules can be incorporated into the matrix material by diffusion at a temperature, close to the matrix melting temperature (firing) [4]. To avoid oxidation, firing often must take place in inert atmosphere. Often, as is the case for BaFBr:Eu^2+^, also the host material is formed in the firing process by reaction between raw materials. BaFBr can be made in a solid state reaction by firing a mixture of BaF_2_ and BaBr_2_ or by a solid-gas reaction between BaF_2_ and NH_4_Br or between BaBr_2_ and NH_4_F. Since the phosphor particle size is an important quality parameter, milling may be an essential step in powder phosphor manufacturing. The first step in PIP manufacturing is making a lacquer, based on phosphor powder. A polymer binder is dissolved in a solvent (mixture). Next, the phosphor powder is dispersed in the solution, which gives a thick, white, phosphor “paint”. This “paint” is coated on a substrate (often a PET film) and the solvents are evaporated. The dried layer consists of phosphor particles, held together by a binder. Since the phosphor layer is vulnerable and must be cleanable with liquid it is covered with a scratch-resistant protective coating (Figure 7).

### 3.2. The BaFBr:Eu^2+^ Storage Phosphor

**Figure 8:**
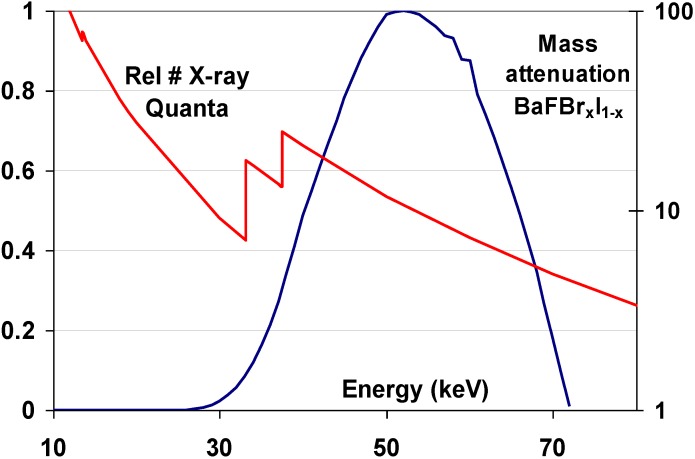
Overlay of the mass attenuation coefficient of BaFBr_0.85_I_0.15_ and a typical general radiography X-ray spectrum.

At the time CR was invented a sufficiently performant storage phosphor was lacking. BaFCl:Eu^2+^ and BaFBr:Eu^2+^ had been of the first rare-earth X-ray phosphors for screen/film radiography [5]. The BaFX matrix is a layered material with a tetragonal PbFCl or Matlockite structure [6]. The Eu activator substitutes Ba for a small amount. Even today, BaFBr:Eu^2+^ is still in use in intensifying screens. Originally, however, the BaFX:Eu^2+^ phosphors were suffering from a high degree of after-glow [5]. This indicates that, after X-ray exposure, charges are trapped in the phosphor that slowly leak away to give rise to delayed luminescence. In hindsight, it is no surprise, therefore, that the most important phosphors for medical CR are of the BaFX:Eu^2+^ family. It was at the end of the seventies that Fuji discovered that BaFBr:Eu^2+^ is an excellent storage phosphor for use in medical radiography [7]. It has a density of about 5 g/cm^3^, a K-edge of 37 keV that is well suited for general radiography (Figure 8) and the Eu^2+^ excited state lifetime of about 800 nsec allows sufficiently fast scanning in a CR digitizer. The discovery of this material started the development of CR and a cascade of patents followed. It took about 15 years to develop a market ripe product. CR was introduced around 1985 [8,9] by Fuji and found entrance on a significant scale in the 90’s of the previous century. In the period from 1992 to 1994 also Agfa, Kodak and Konica launched CR.

## 4. BaFBr:Eu^2+^ PSL Mechanism

As was pointed out, X-rays generate electrons and holes that can move freely through the phosphor lattice. When a significant portion of these charge carriers is trapped in lattice defects, and when these traps are stable at room temperature, the phosphor is capable of storing X-ray images. Storage and read-out processes are shown schematically in Figure 2 and Figure 3. Usually, the trapped electron is photo-excited and recombines with the trapped hole. The recombination energy is transferred to an electron of a luminescent impurity center, in the phosphor crystal lattice: the activator. When the activator electron returns to the ground state a photon is emitted in the PSL process.

In BaFBr:Eu^2+^ the activator, obviously, is Eu^2+^. The different theories proposed for explaining energy storage and release will now be highlighted.

**Figure 9:**
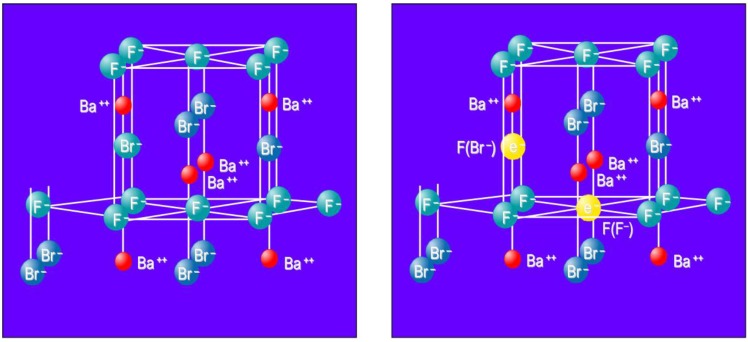
Model of the BaFBr lattice cell (**a**) with F(Br^−^) and F(F^−^) centers (**b**).

From the start it was claimed that X-rays generate F centers as electron traps. An F center is an electron trapped in an anion vacancy. In the Matlockite structured BaFBr, Ba^2+^ layers are alternately interspaced by two Br^−^ layers and single F^−^ layer (Figure 9a). Hence, F(Br^−^) and F(F^−^) centers are created as electron traps (Figure 9b).

In [10], it is shown that both centers are generated and contribute to PSL. However F(F^−^) centers are only generated above 200 K, and the generation process has not yet been understood [11]. For practical stimulation with a HeNe laser (633 nm) or diode lasers (650 nm–680 nm) the contribution of F(F^−^) centers to PSL is negligible [12]. Nevertheless, the stimulation spectrum of BaFBr:Eu^2+^ has two stimulation bands. The reason is that the F center has tetragonal symmetry in BaFCl and BaFBr. For the electrical vector of the stimulation light E ⊥ c axis, the F(Br^−^) absorption has its maximum at about 590 nm, and for E // c axis at about 500 nm in BaFBr [13]. The absorption ratio is due to the statistical distribution of parallel and perpendicular crystal orientation wrt. the stimulation light electrical vector. A similar observation had been made earlier for BaFCl [6].

### 4.1. Electron and Hole Center Production

A first mechanism for the PSL process was proposed by Takahashi [9,14]. It is based on the assumption that Br^−^ vacancies are present within the virgin lattice. The electrons are trapped in the vacancies by which F(Br^−^) centers are created. It is, further, assumed that the holes are trapped by Eu^2+^ ions, which are thus oxidized to Eu^3+^ (Equation (1)). Takahashi argues that the F center electrons enter the conduction band and recombine with Eu^3+^ ions upon photo stimulation of the F(Br^−^) center. Recombination results in an excited Eu^2+^ ion (4f^6^5d^1^ state), that decays emitting the characteristic 390 nm luminescence.

(1) $$\begin{array}{l} V_{Br_{-}}\;\overset{\;X-ray\;}{\to }F(Br^{-})\\ {\text{Eu}}^{2+}\overset{\;X-ray\;}{\to }{\text{Eu}}^{3+} \end{array}$$

An alternative mechanism proposed for alkali halides [15] was adopted by von Seggern *et al*. for BaFBr:Eu^2+^ [12]. According to this model, X-rays generate excitons which upon decay may displace a Br^−^ ion from its lattice position leaving the electron behind, *i.e.*, forming an F(Br^−^) center. The Br atom then combines with a Br^−^ ion at an adjacent lattice position. A so-called H center is created: a negatively charged halide molecule that occupies a single halide lattice position (Equation (2)). In this model there is no need to assume that anion vacancies are present in the virgin material. Upon F center photo stimulation the released electron may recombine with the H center hole trap. This excites the Eu^2+^ activator, resulting in PSL.

(2) $$\begin{array}{l} h\upsilon \;\overset{\;X-ray\;}{\to }(e^{-}/h)exciton\\ \left(e^{-}/h)exciton\overset{\;X-ray\;}{\to }F(Br^{-})+H(Br_{2}^{-}\right) \end{array}$$

However, none of the scientific groups that investigated the BaFBr:Eu^2+^ PSL mechanism has been able to demonstrate an increase in Eu^3+^ concentration or the presence of H centers after X-ray exposure. The lack of experimental support for the described models gave rise to a third proposal for the formation of defect centers by Spaeth *et al.* [16,17,18,19,20]. This model equally assumes that vacancies are already present in the virgin lattice. The origin of the Br^−^ vacancies in BaFBr is discussed in 4.4. Spaeth *et al*. proved experimentally that X-ray exposure of BaFBr single crystals results in the formation of F(Br^−^) and V_K_ center pairs.

(3) $$V_{Br^{-}}\overset{\;X-ray\;}{\to }F(Br^{-})+V_{K}(Br_{2}^{-})$$

**Figure 10:**
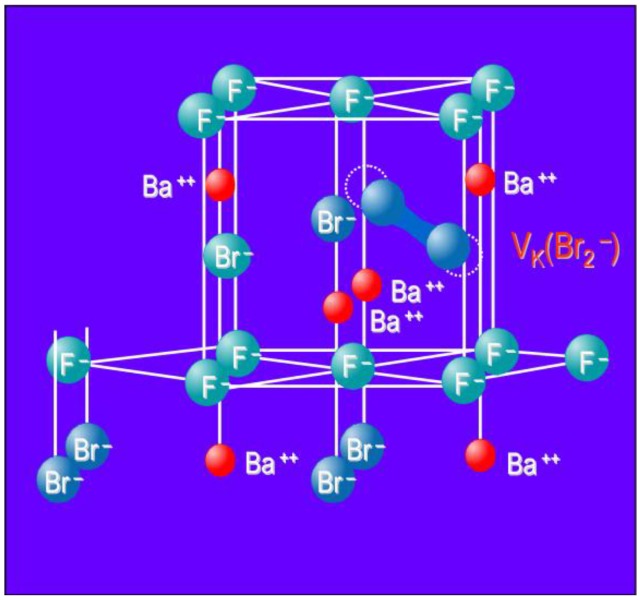
Model of the BaFBr lattice cell with V_K_ (Br_2_^−^) center.

The electron is trapped in an anion vacancy and the holes by two adjacent Br^−^ lattice ions. As a result both Br^−^ ions shift slightly towards each other into the interstitial space. The resulting Br_2_^−^ dumbbell is a called a V_K_ center (Figure 10). Recombination of the F(Br^−^) electron with a V_K_ center upon photo stimulation releases energy that excites the Eu^2+^ activator resulting in PSL. Although the hole trap has not yet been identified spectroscopically, this model is until now not contradicted by experiment.

### 4.2. Recombination of Trapped Electrons and Holes

After excitation of the F center electron the F center environment undergoes a lattice relaxation due to the different charge distribution in the ground and excited states [21,22,23]. The energy of the electron in the relaxed excited state (RES) is very close to that of the conduction band. For release into the conduction band a thermal energy of 35 meV is needed [21]. Hence, the probability for escape into the conduction band is determined by the lattice temperature. 

Alternatively, the electron may tunnel to the hole center. Only for centers separated by a few lattice constants this process has non-zero probability. In case neither of the above processes happens during the intrinsic lifetime of the RES, the electron falls back to its ground state and emits an IR photon with 0.9 eV energy.

Electron transport via the conduction band can be ruled out as charge transfer process and tunneling should be considered as the only possibility. The decay of PSL under continuous stimulation is temperature independent [24]. If the PSL process would require thermal energy to inject the electrons into the conduction band this would clearly not be the case. Also efficient PSL has been detected down to temperatures of 1.5 K. This cannot be explained by a model that relies on electron transport via the conduction band.

In order to transfer the recombination energy to the Eu^2+^ activator the electron and hole traps should be spatially correlated with it. For efficient PSL the electron and hole traps need to be spatially correlated with each other (tunneling probability) and with the Eu^2+^ activator (energy transfer). Hence, triple defect aggregates are required for efficient PSL. Within such a triple aggregate the distances between the individual defect centers should be only a few lattice constants.

### 4.3. Spatial Correlation of Defects

The electron-hole pair concentration resulting from exposure to a medical X-ray dose is extremely low, *i.e*., <10^13^ per cm^3^. The concentration of F/V_k_ centers will be even lower as a significant portion of the electron hole pairs will recombine before being trapped. The activator concentration is approximately four orders of magnitude higher *i.e.*, 10^17^ per cm^3^, but this is still very low .These low concentrations make it very unlikely that the F(Br^−^)/V_K_ center pair will be created in the vicinity of a Eu^2+^ ion. 

Nevertheless, efficient PSL is still observed, at 1.5 K. At this temperature the mobility of the F and V_K_ centers is practically zero. The measured PSL at 1.5 K indicates that triple aggregates are already formed at these low temperatures. Experimentally a spatial correlation between F/V_K_ centers and Eu^2+^ was indeed found [19,25]. Although it has not strictly been proven, one may assume that the exciton decay causing the formation of the F/V_K_ centers preferentially occurs in the vicinity of the Eu^2+^ activator site.

Initially, the F(Br^−^)/V_K_ center pair is spatially correlated but at room temperature the centers may diffuse away from each other, since the V_K_ center is mobile above 120 K, and the F(Br^−^) center above 200 K [19,20]. 

At higher temperatures the formation of triple aggregates is assumed to be a dynamic equilibrium. As a consequence of the defect mobility, some aggregates will be broken while others will be formed. This assumption is supported by the replenishment effect. At low temperature, a phosphor crystal is stimulated until it is exhausted. When it is next heated to a temperature above 200 K, it can be stimulated again [15].

### 4.4. The Formation of Bromine Vacancies in the BaFBr Lattice

BaFBr powders produced by firing stoichiometric ratios of BaF_2_ and BaBr_2_ or single crystals grown from a melt of such mixtures are all contaminated with oxygen, probably because of the hygroscopic nature of BaBr_2_. Spaeth *et al*. demonstrated experimentally that oxygen is incorporated in the fluoride sub lattice, substituting for F^−^, *i.e*., forming O_F_^2−^ centers. For charge compensation an anion vacancy is needed. This anion vacancy proved to be a Br^-^ vacancy [11,17,18].

**Figure 11:**
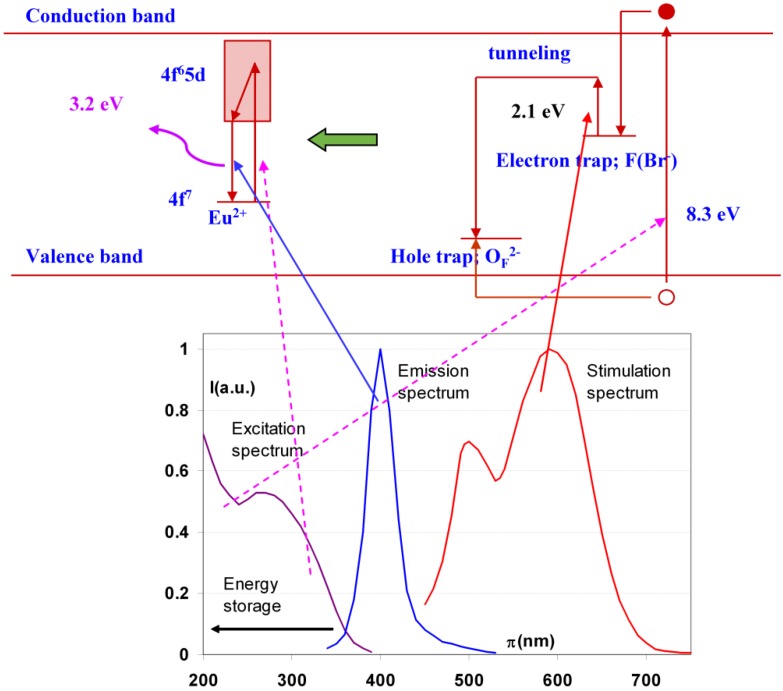
Energy diagram showing photo-stimulated luminescence in BaFBr:Eu^2+^ and the relation with the stimulation, excitation and emission spectrum of the material.

Above 120 K the O_F_^2−^ center may react with the V_K_ center to form a new hole center O_F_^−^ (Figure 11).

(4) $$\begin{array}{l} O_{F}^{2-}\;+V_{{\text{Br}}^{-}}\;\;\;\overset{\;X-ray\;}{\to }V_{K}(Br^{-})+F(Br^{-})+O_{F}^{2-}\\ O_{F}^{2-}+V_{K}(Br^{-})\overset{\;X-ray\;}{\to }F(Br^{-})+O_{F}^{-} \end{array}$$

## 5. BaFBr:Eu^2+^ Engineering

Storage phosphors used in CR depend on efficient trapping and storage of free electrons and holes. Hence, changing the storage center characteristics has been the subject of most of BaFBr:Eu^2+^ storage phosphor engineering. The Mollwo-Ivey relation, based on the particle-in-a-box model, relates the peak F center absorption wavelength to the defect size: the absorption wavelength is proportional to d^1.84^, where d is the nearest neighbor distance [26]. The model was derived for a vacancy with cubic symmetry, but it can be used to explain in a qualitative way the impact of lattice changes on F-center characteristics. Partial or complete replacement of Ba and/or Br in the BaFBr lattice changes the F center dimensions and the stimulation spectrum of the storage phosphor. One particular change of the phosphor composition produced a change in the nature of the hole trap.

**Figure 12:**
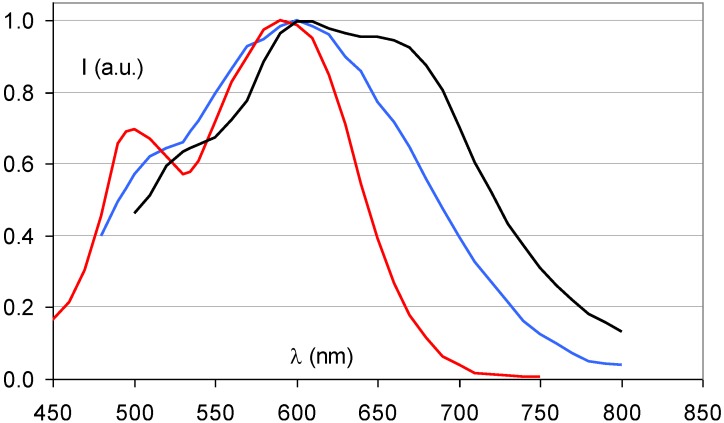
Stimulation spectra of BaFBr_0.85_I_0.15_:Eu^2+^ (blue), BaFI:Eu^2+^ (black) (data from [27]) and BaFBr:Eu^2+^ (red).

In the course of the storage phosphor development it was discovered that partial replacement of Br^−^ by I^−^ almost doubled the storage phosphor efficiency. In the commercial phosphor of Agfa, Fuji and Kodak 15 to 20% of Br^-^ is replaced by I^−^. I^−^ (2.20 Å) having a larger size than Br^−^ (1.96 Å) this modification not only increases the storage capacity. It also leads to lattice expansion, which, as explained above, causes a red-shift of the stimulation spectrum (Figure 12). The additional benefit I^−^ incorporation is, therefore, that smaller, cheaper and more efficient semiconductor diode lasers emitting in the 650 to 700 nm wavelength region can be used as light source for read-out in CR scanners instead of a He-Ne laser. 

A logical further modification is the complete replacement of Br^−^ by I^−^, *i.e*., the transition to BaFI:Eu^2+^. This is exactly what has been proposed by Nakano *et al*. [27,28]. Replacement of Br^−^ by I^−^ leads to a higher crystal density. In combination with the higher atomic number of iodide and its K-edge at about 35 keV this leads to a higher specific X-ray absorption, especially for general radiography exposures. A disadvantage is the much higher hygroscopicity of BaFI. Much stronger efforts must be made to shield the phosphor from moisture. The further expansion of the crystal lattice by complete replacement of Br^−^ by I^−^ results in an even stronger red-shift of the stimulation spectrum (Figure 12).

**Figure 13:**
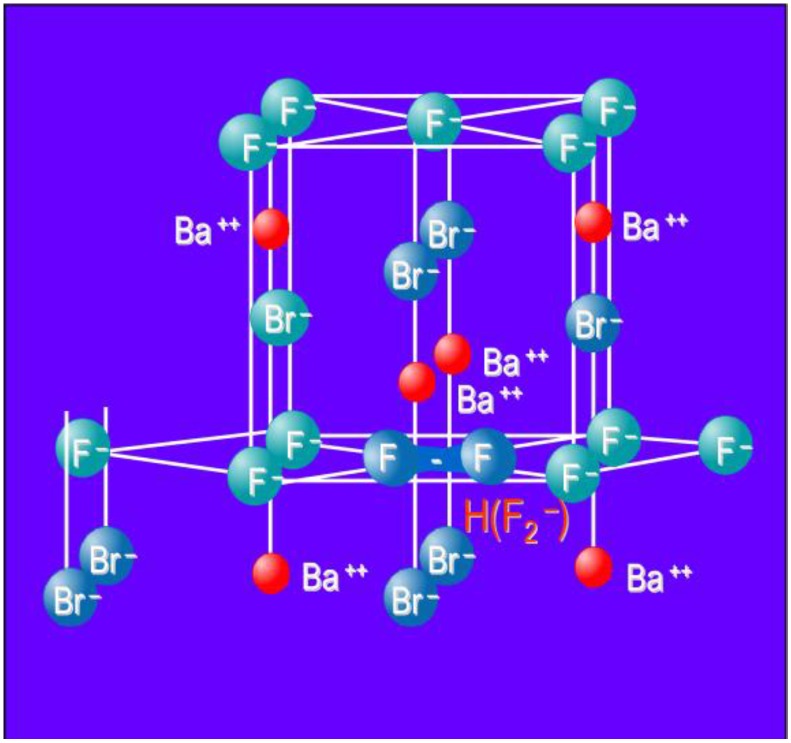
Model of the BaFBr lattice cell with H (F_2_^−^) center.

In the initial CR development period, Agfa was faced with a pile of Fuji patents on the BaFX:Eu^2+^ (X = Br^−^, I^−^) CR phosphor. To come up with an alternative it developed a close relative, in which a significant fraction of Ba is replaced by Sr and in which F^-^ is in excess of Br^-^, Ba_1−x_Sr_x_F_1+y_X_y_:Eu^2+^ (x > 0.07; y > 0.05) [29]. The most significant difference proved to be the F-excess. This excess can only be realized by using BaF_2_ and NH_4_Br as starting materials for the phosphor production in a solid-gas reaction. Klee demonstrated that a phosphor with an excess of F^−^ as high as 10 ± 2% could be made, still having the Matlockite structure and without having more than 1% of second phase contaminants [30]. Since in such a phosphor, no oxygen luminescence could be detected [31], it was concluded that the material is oxygen-free in contrast to stoichiometric BaFBr:Eu^2+^, in which oxygen plays an essential role in the PSL process. The fact that no F(F^−^) centers could be detected after X-ray exposure and that the density of the material was very similar to that of stoichiometric material lead to the speculation that the non-stoichiometric phosphor has F^−^ antisites, *i.e.*, F^−^ on Br^−^ sites. The existence of such antisites was established by magic angle spinning nuclear magnetic resonance (MAS-NMR), a technique that allows distinguishing between F^-^ on regular sites and on Br^−^ sites [32,33]. An EPR study lead Schweizer *et al*. to the conclusion that PSL in the non-stoichiometric phosphor is based on a new hole trap, different from the O_F_^2−^ center that is converted to O_F_^−^ upon hole trapping in stoichiometric BaFBr:Eu^2+^. It was established that X-ray exposure created besides the expected F(Br^−^) centers as electron traps F_2_^−^ molecular centers or H-centers in the F^−^ sub lattice (Figure 13). Hence, charge separation in the non-stoichiometric phosphor is realized by the process of electron trapping in the Br^−^ sub lattice and hole trapping in H centers in the F^−^ sub lattice.

**Figure 14:**
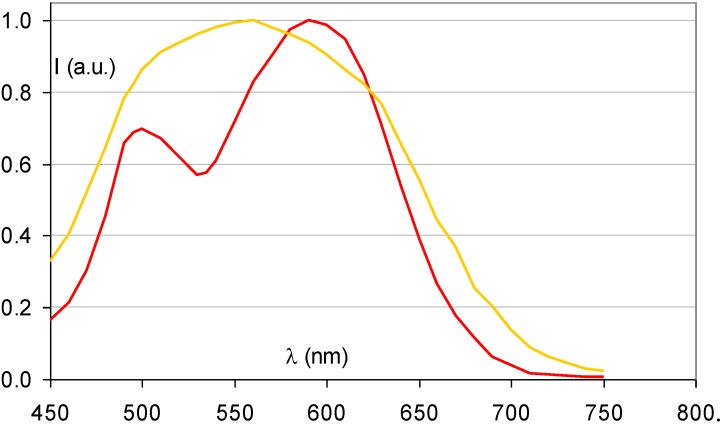
Stimulation spectrum of Ba_0.82_Sr_0.18_FBr:Eu^2+^ (gold) (data from [34]) and BaFBr:Eu^2+^ (red).

**Figure 15:**
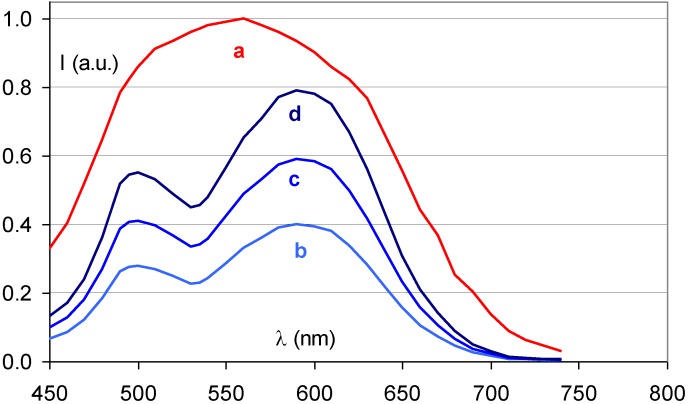
Stimulation spectrum measured at 300 K after X-ray exposure of Ba_0.88_Sr_0.12_F_1.09_Br_0.91_:Eu^2+^ (**a**), and after erasure and 2 (**b**), 4 (**c**) and 8 (**d**) h replenishment.

Figure 14 demonstrates that the partial replacement of Ba^2+^ by Sr^2+^ causes a broadening of the stimulation spectrum. This has been attributed to an inhomogeneous distribution of Sr^2+^ and Ba^2+^ neighbors around the F centers [34]. F centers neighboring an impurity ion are called F_A_ centers and their absorption spectrum is influenced by the presence of the foreign ion. The F_A_(Br^−^) centers in Ba_0.82_Sr_0.18_F_1+x_Br_1−x_:Eu^2+^ have a Sr^2+^ neighbor with a size of 1.12 Å replacing a Ba^2+^ ion with 1.34 Å size. They are larger than F(Br^−^) centers, which explains the red-shift of a part of the stimulation spectrum. A study by Batentschuk *et al*. [35] further demonstrated that Sr doping not only gives rise to F_A_(Br^−^) center formation, but that it also increases the PSL-response in the bands ascribed to F(Br^−^) centers. The increased PSL response has been explained by the fact that the Sr^2+^ ions stabilize the hole centers at room temperature in a similar way as Eu^2+^ [36]. F_A_(Br^−^) centers seem to be less stable than unperturbed F(Br^−^) centers. The red-shift of the stimulation spectrum disappears upon thermal activation above room temperature [37]. The thermally activated change of the stimulation spectrum is also observed when in the so-called “replenishment” effect in Ba_0.82_Sr_0.18_F_1+x_Br_1−x_:Eu^2+^ (Figure 15). After X-ray exposure and exhausting read-out, the stimulation spectrum gradually recovers to some extent when the electrons that are still trapped, redistribute themselves over the trapping centers. The recovered stimulation spectrum is that of stoichiometric BaFBr:Eu^2+^.

**Figure 16:**
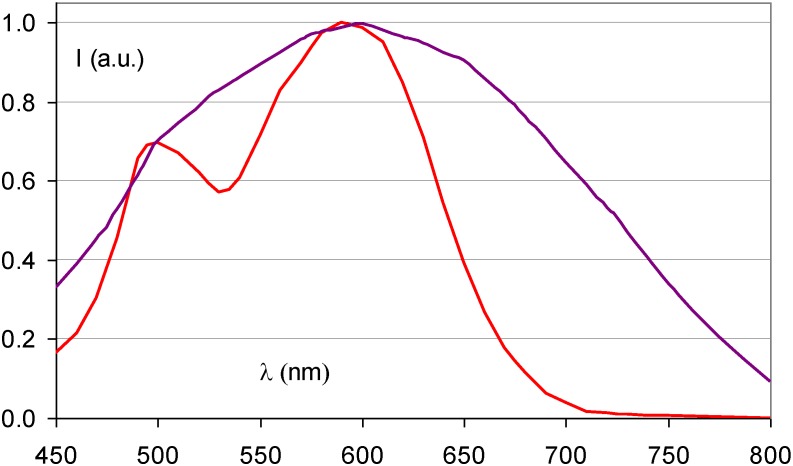
Stimulation spectra of Ba_0.99_Ca_0.01_FBr:Eu^2+^ (purple) (data from [39]) and BaFBr:Eu^2+^ (red).

Ca^2+^ Incorporation has a similar effect as Sr^2+^ incorporation (Figure 16). It results in a broadened stimulation spectrum due to the presence of a Ca^2+^ induced F_A_(Br^−^,Ca^2+^) center with stimulation maxima at 540 and 680 nm [38]. It was shown that the X-ray sensitivity, *i.e*., the number of storage centers formed per unit of absorbed X-ray energy, increases up to a doping level of 1 mole%. In addition, it was shown that F_A_ centers are formed far beyond the statistical probability corresponding to the Ca^2+^ doping level. The estimated ratio of F_A_ to unperturbed F centers was approximately 3:1 for a doping level of 2%. The sensitivity drops rapidly at higher concentrations of Ca, probably because the much smaller Ca^2+^ ions (size = 0.99 Å) can replace Ba^2+^ only to a small extent without bringing too much stress in the BaFBr lattice.

**Figure 17:**
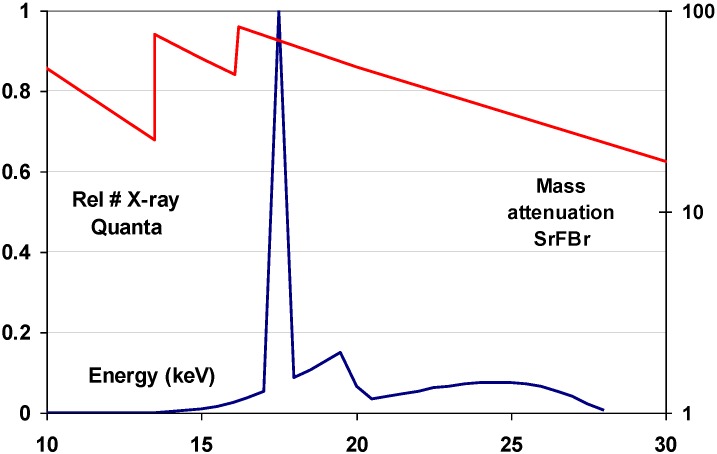
Overlay of the mass attenuation coefficient of SrFBr and a typical mammography X-ray spectrum.

The 37.4 keV Ba K-edge is well suited for high specific X-ray absorption in general radiography, where X-ray spectra are used having a maximum at about 50 keV. A typical mammography X-ray spectrum, however, is in the range from 15 to 30 keV (Figure 17).

**Figure 18:**
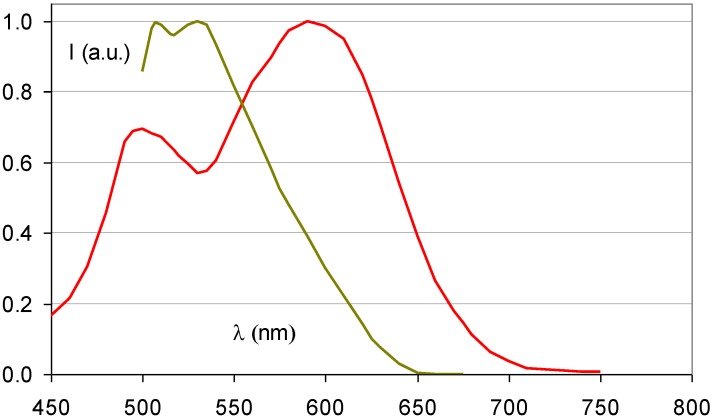
Stimulation spectrum of SrFBr:Eu^2+^ (green) (data from [40]) and BaFBr:Eu^2+^ (red).

As a consequence, SrFBr:Eu^2+^ containing Sr with its K-edge at 16.1 keV is more suited for this application. As a matter of fact, in spite of its slightly lower density of 4.7 g/cm^3^ and in spite of the lower atomic number of Sr, SrFBr:Eu^2+^ has a higher specific X-ray absorption than BaFBr:Eu^2+^ in the range of 16.1 to 37.4 keV (Figure 17). Hosoi *et al*. demonstrated that the Sr-based phosphor leads to very good image quality in mammography [40]. Due to the smaller cation size, SrFBr has smaller lattice constants than BaFBr. This entails smaller F-centers after X-ray exposure requiring higher energy to release the trapped electrons in the read-out process. Efficient stimulation requires light in the 500–550 nm, instead of the 570–630 nm range (Figure 18). At the time of the investigation lasers emitting in this range showed poor stability and were much more expensive than lasers emitting in the 600–700 nm range. Another disadvantage is that SrFBr:Eu^2+^ is much more hygroscopic than BaFBr:Eu^2+^. Hence, a much larger effort is needed to stabilize the IP’s. This may explain why the material never made it to the commercial stage.

## 6. Alternative Storage Phosphors for Medical CR

BaFX:Eu^2+^ (X = Br, I) complies remarkably well with all CR phosphor requirements. There were two main motives to search for alternatives. The first was simply the pile of Fuji patents on this CR phosphor family. The second was an evolution in radiographic 2D medical imaging. A newer and promising imaging technology, direct radiography (DR) based on flat panel arrays, was launched in the mid-nineties. Since, at that point, this technology allowed a lower patient dose compared to CR, it was a challenge to make CR more dose efficient. Translated to storage phosphor characteristics this means higher X-ray absorption and more light output for the same absorbed dose. This can be realized by using a different phosphor compound with a higher specific X-ray absorption, or, alternatively, by making the phosphor layer thicker without compromising spatial resolution. Thicker phosphor coatings can be realized using phosphor compounds that can be grown into needles. Needle phosphor will be dealt with in paragraph 7. Another way to manufacture a useful thicker phosphor layer is by using semi-transparent storage phosphor glass ceramics.

This overview shortly deals with:

- the most promising candidates to replace BaFBr:Eu^2+^
- storage phosphors having high X-ray absorption or useable for manufacturing thicker and, therefore, more absorbing phosphor screens.

It was not intended to be complete.

Storage phosphors are compared based on the following quality parameters [41]:

Conversion efficiency (CE): the total energy of stimulated light per unit area and per unit of X-ray dose produced by the phosphor in pJ/mm^2^/mR,
Stimulation energy (SE): the laser energy per unit area required to release 63% of the stored energy in µJ/mm^2^.

Often the Figure of Merit (FOM) is used as an overall quality measure of a storage phosphor. This only makes sense, however, when phosphors are compared that have stimulation energies in the same range. 

### 6.1. BaFBr:Eu^2+^ Alternatives

Bariumbromosilicate, Ba_5_SiO_4_Br_6_:Eu^2+^, was first synthesized and characterized by Blasse *et al.* [41,42] in 1989. The material was doped with Eu^2+^ and co-doped with a trivalent rare earth element from the group: Y, La, Gd and Lu. The stimulated emission peaks at 440 nm. The stimulation spectrum consists of two stimulation bands: one with a maximum below 500 nm and one with a maximum at 610 nm. Stimulation with green light (500 nm ≤ λ ≤ 550 nm) is approximately twice as efficient as stimulation with red light from a He-Ne laser (λ = 633 nm). The SE was 40 μJ/mm^2^ for green stimulation and 70 μJ/mm^2^ for red stimulation, respectively. Since the SE of BaFBr:Eu^2+^ is of the order of 15 μJ/mm^2^ higher laser power is needed for stimulation of this phosphor. The CE of the storage phosphor was of the same order of magnitude as that of the contemporary BaFBr:Eu^2+^ reference, *i.e*., 3.8 pJ/mm^2^/mR. At this level, however, that the X-ray energy needed to create a stimulable defect center is still 6 to 7 times higher than for optimized BaFX:Eu^2+^ phosphors. An advantage of bariumbromosilicate resides in the fact that it is much less hygroscopic than BaFBr:Eu^2+^. Ba_5_SiO_4_Br_6_:Eu^2+^ phosphors were also optimized for IR stimulation and were patented for this [43]. Based on the level of the PSL properties, however, Agfa decided to develop Ba_0.82_Sr_0_.18F_1+x_Br_1−x_:Eu^2+^ for commercial use and not bariumbromosilicate.

Storage properties of rare earth doped phosphates were investigated by Blasse *et al*. [44,45]. Ba_3_(PO_4_)_2_:Eu^2+^ powder phosphor was made together with the co-doped material Ba_3_(PO_4_)_2_:Eu^2+^,La^3+^. All samples exhibited PSL with an emission peaking at 410 nm. The stimulation spectrum peaks at about 475 nm but has a tail extending to 800 nm. It was found that co-doping with a trivalent ion enhanced the CE. The first non-optimized samples showed a promising storage capacity: the CE of the co-doped sample was 20% of that BaFBr:Eu^2+^. The main disadvantage was the SE that was much higher than that of BaFBr:Eu^2+^: 5 mJ/mm^2^ *versus* 15 μJ/mm^2^. This was attributed to the low absorption strength of the trap or to the low re-combination probability.

### 6.2. Higher Intrinsic Absorption

With its density of 9.4 g/cm^3^ terbium doped Lu_2_O_3_, Lu_2_O_3_:Tb^3+^, was a candidate storage phosphor with higher intrinsic absorption. A sintered ceramic of this material was demonstrated to have storage phosphor properties [46]. The PSL has the strongest lines between 540 and 560 nm. It can be stimulated either with IR (980 nm) or with a red (647 nm) light. The red stimulation is more efficient but is more difficult to separate from the stimulated emission. The emission wavelengths of Lu_2_O_3_:Tb^3+^, clearly, do not match well with the conversion efficiency spectrum of a PMT.

### 6.3. Elpasolite Storage Phosphor

Cs_2_NaYF_6_:Ce^3+^ is a compound with a cubic elpasolite structure that has storage phosphor capacities and that can be grown into needles [47]. It was patented by Agfa [48]. PSL peaks at 360 nm and the stimulation spectrum at 550 nm. Therefore, it must be excited with a green laser, as, e.g., a frequency doubled Nd:YAG laser, emitting at 532 nm. These lasers are more expensive than red diode lasers with the same power. When excited at 532 nm the conversion efficiency is 15 pJ/mm^2^/mR and the stimulation energy 90 μJ/mm^2^. Fading of the stored energy is still acceptable: approximately 30% is lost in the first hour [47].

### 6.4. Alkaline Earth Sulfide Storage Phosphors

Rare earth doped alkaline earth sulfides are efficient scintillators. Chakrabarti [49,50] showed that MgS:Ce^3+^:Sm^3+^, MgS:Eu^2+^:Sm^3+^ and CaS:Ce^3+^:Sm^3+^ also exhibit storage phosphor characteristics. The low X-ray absorption of these materials disqualifies them as medical detector materials. However, they are well suited for dosimeter applications.

Intense PSL was also found in SrS:Eu^2+^:Sm^3+^ [51], a compound with acceptable X-ray absorption for mammography. The stimulated emission peaks at 600 nm, and the stimulation spectrum extends from 800 nm to 1700 nm, with a peak close to the wavelength of a Nd:YAG laser (1.06 µ). 

A similar material, SrS:Ce^3+^:Sm^3+^, was patented and commercialized by Liberty Technologies [52]. This material showed a stimulated emission at 485 nm. The stimulation spectrum shows peaks at 590 and 970 nm. The yellow peak (590 nm) is approximately twice the size of the IR peak (970 nm).

What is interesting about this class of storage phosphors is that a “redox couple” of rare earths is used for energy storage and PSL. One of the rare earths serves as hole trap; the other as electron trap. For example, following charge transitions are believed to take place in SrS:Ce^3+^;Sm^3+^:

(5) $$\begin{array}{l} Ce^{3+}\;+{\text{Sm}}^{3+}\;\overset{\;X-ray\;}{\to }\;Ce^{4+}+Sm^{2+}\\ Ce^{4+}+Sm^{2+}\overset{\;PSL\;}{\to }{\text{Ce}}^{3+}+Sm^{3+} \end{array}$$

A general approach for the development of storage phosphors, based on this principle, has been proposed by Dorenbos. Empirical models have been developed that predict accurately the location of lanthanide energy levels within the forbidden gap of inorganic compounds. These models have been used to locate the 5d excited and 4f ground states relative to the conduction and valence band of host compound [53]. A suitable electron trapping lanthanide can be selected and combined with a suitable recombination center. This makes it possible to control what lanthanides are storing what type of charge carrier and how deep they are stored. Deliberate design of lanthanide based electron storing phosphors is achievable in this way and might lead to alternative classes of storage phosphors.

### 6.5. Storage Phosphor Glass Ceramics

Bromine and europium co-doped fluorobromozirconate glass ceramics exhibit weak PSL. This effect is strongly enhanced after thermal annealing [54]. The glasses contain 20% of BaF_2_. Annealing results in BaBr_2_ nano-crystals that are incorporated in the glass material. Hence the designation glass ceramic. After annealing the transparent glass is transformed into a semi-opaque material. This is due to a phase transition of the metastable hexagonal BaBr_2_ nano-crystals to larger stable orthorhombic BaBr_2_ crystals. These crystals exhibit more efficient PSL properties. The stimulated emission has its maximum at 404 nm and the stimulation spectrum at 580 nm.

PSL efficiency was reported to be 9% of that of the BaFBr:Eu^2+^ reference. When correction is made for the small fraction that the crystallites constitute (≅7%), the PSL is nearly 1.3 times that of BaFBr:Eu^2+^.

## 7. Needle Storage Phosphors for Medical CR 

### 7.1. Needle Plate Manufacturing

An alternative technology to produce phosphor layers is through physical vapor deposition (PVD).

**Figure 19:**
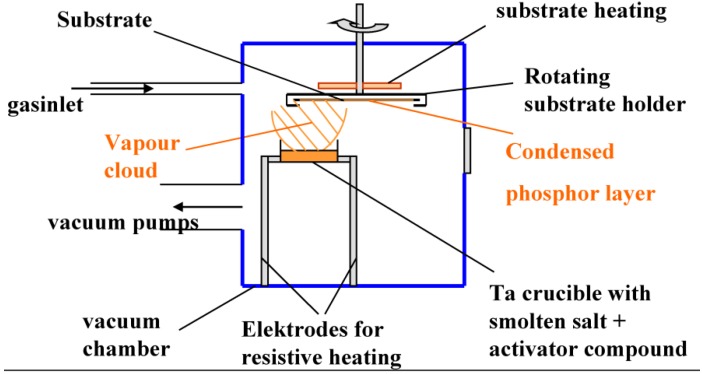
Schematic drawing of a thermal vapor deposition unit for NIP manufacturing.

The most common PVD technique is thermal vapor deposition, but deposition via electron beam sputtering is equally possible. In thermal vapor deposition, the phosphor raw materials, mostly a simple salt and an activator compound, containing the luminescent element, are heated to above the melting temperature in a vacuum chamber. This happens by resistive heating of metal crucibles containing the raw materials. In vacuum a gas cloud is produced above the liquid salt, which then condenses on the colder substrate suspended above the crucible (Figure 19).

**Figure 20:**
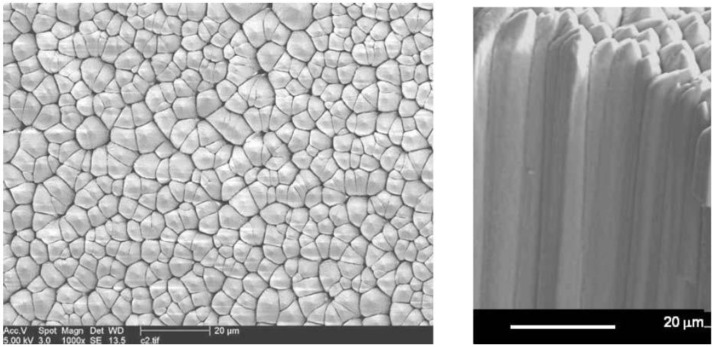
Top and side SEM images of a needle crystalline phosphor layer.

A crystalline layer of phosphor is deposited on the substrate. In a certain pressure and substrate temperature range, phosphor crystals grow as needles, perpendicular to the substrate surface [55,56,57] (Figure 20).

Since materials for vapor deposition must melt congruently (without decomposition), needle phosphors are mostly chemically simple compounds, containing 1 anion and 1 cation.

Vapor deposited needle imaging plates (NIP’s) have a number of advantages over PIP’s. They have lower self-absorption of emitted light leading to higher sensitivity. Lower self-absorption also allows the use of thicker layers, having higher X-ray absorption. Higher X-ray absorption also results from a higher packing density. In addition, suppression of lateral light diffusion in a vapor deposited layer leads to improved resolution [5,58] (see also Paragraph 9.3.2).

### 7.2. Needle Phosphors for Medical Imaging

CsI:Na phosphor single crystals were first reported on by Brinkman [59]. This phosphor has an excellent intrinsic X-ray absorption, being made up of Cs with a K-edge of 36.0 keV and I with a K-edge of 33.2 keV. CsI:Na vapor deposited layers were proposed as input phosphor layers for image intensifiers by Bates [60] in 1969. Needle layers were introduced for these imagers in the subsequent period. Image intensifiers are dynamic X-ray imagers used, for example, to visualize the heart beat of a patient [61]. The CsI:Na needle layer transforms the X-ray image directly into a light image, which then generates an electronic image in a photocathode. In image intensifiers the CsI:Na layer is enclosed in a vacuum tube. Vapor deposited CsI:Na would also be an excellent phosphor for screen/film radiography, leading to much better image quality than even rare-earth phosphors like BaFCl:Eu^2+^ and Gd_2_O_2_S:Tb^3+^ [62]. However, the high moisture sensitivity makes it unsuitable for this application. It had been demonstrated by Kano *et al*. [63] that CsI:Na can be an efficient X-ray storage phosphor, having adequate spectroscopic properties with a blue emission and a stimulation spectrum with a maximum between 700 and 800 nm. However, on top of its very hygroscopic character the material must be cooled for energy storage, because the trapped holes escape to migrate and recombine with the trapped electrons far below RT.

CsI:Tl is much less hygroscopic than CsI:Na. However, the phosphor’s broad emission band, centered at about 590 nm does not match well with standard S11 photocathodes or with standard X-ray films [62]. Therefore, it has never been used in image intensifiers or in film/screen systems. It was, however, selected for use in direct radiography (DR) flat-panel detectors [64]. Here, the X-ray image is transformed into a light image, to then generate charges in an a-Si photodiode layer. The emission matches very well with the absorption spectrum of the a-Si photodiode layer [65]. However, since free charge carrier trapping is negligible in CsI:Tl it cannot be used in CR.

### 7.3. CR Needle Phosphors

**Figure 21:**
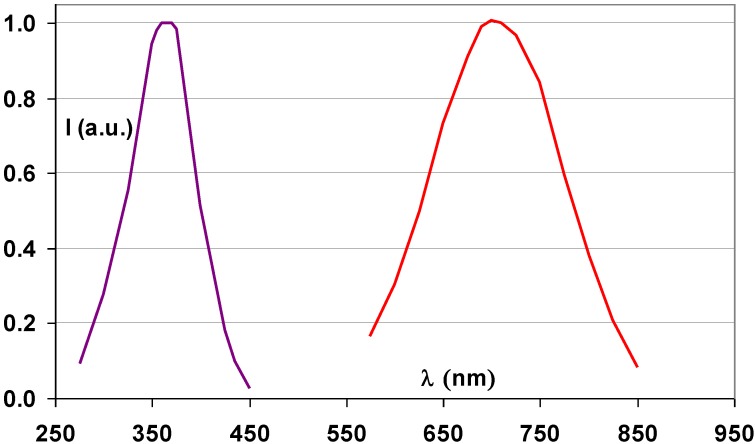
Emission (violet) and stimulation (red) spectrum of RbBr:Tl^+^ storage phosphor (data from [67]).

Efforts were made to deposit BaFBr:Eu^2+^ storage phosphor films by evaporating mixtures of BaF_2_ and BaBr_2_ [66]. BaF_x_Br_1−x_ solid solutions were formed that did not have the luminescent efficiencies of the powder phosphor. Konica discovered that RbBr:Tl^+^ is an efficient storage phosphor [67,68] with excellent PSL properties. The 700 nm maximum in the stimulation spectrum allows the use of a semiconductor laser for stimulation (Figure 21). The 0.3 μs decay-time of the PSL is sufficiently short for fast read-out and the amount of light required to release the remaining trapped charges is small. Energy storage and release in RbBr:Tl^+^ was studied in detail [69,70]. The electron traps in this material are Br^−^ F centers. It was demonstrated that Tl is incorporated in the phosphor in the monovalent state. The suggested PSL process is a tunneling from the relaxed excited state of the F center to an excited state of the Tl^2+^ ion, which is the hole trap. The vapor deposited RbBr:Tl^+^ plate has all the described NIP benefits. However, Rb with its K-edge of 15.2 keV has a relatively low intrinsic X-ray absorption. Therefore, the X-ray absorption of the two times thicker—and more expensive—NIP was even slightly below that of the BaFBr:Eu^2+^ PIP. In addition, RbBr:Tl^+^ is much more hygroscopic than BaFBr:Eu^2+^. This made the use of RbBr:Tl^+^ plates in a cassette system, in which the atmospheric conditions cannot be controlled and where the protective coating can be damaged, risky. Konica used the RbBr:Tl^+^ plates only in wall-stands as the Regius^®^ model 330 and 530 “direct digitizer” systems, in which the phosphor plates together with the read-out optics and erasure unit were incorporated in a closed casing [71].

PSL was investigated in several In^+^, and Ga^+^ doped alkali halides [72,73,74]. The characteristics of these materials are given in Table 1.

**Table 1:** PSL characteristics of In^+^ and Ga^+^ doped alkali halide storage phosphors in comparison with standard BaFBr:Eu^2+^.

Phosphor	Stimulating laser wavelengtd (nm)	Peak PSL wavelengtd (nm)	Peak stimulation wavelengtd (nm)	CE pJ/mm^2^/mR	SE μJ/mm^2^
BaFBr:Eu^2+^	633	390	550	20	16
BaFBr:Eu^2+^	680	390	550	14	28
RbBr:In^+^	680	490	700	2	25
RbBr:Ga^+^	680	550	705	6	4
CsBr:In^+^	680	504	700	3	23
CsBr:Ga^+^	680	515	685	6	4

**Figure 22:**
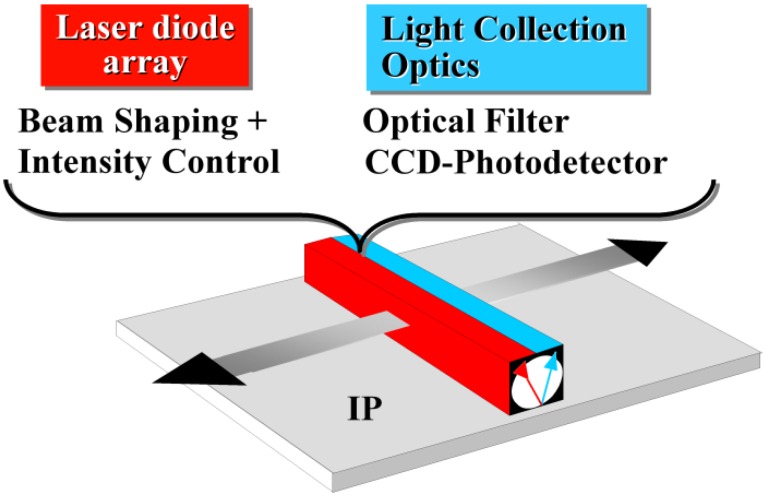
Schematic diagram of line-scanning principle (adapted from [77]).

An interesting feature of these materials is that the peak of the stimulation spectrum practically coincides with the emission of a 680 nm diode laser. The PSL spectrum matches well with the solid state photodiodes. The RbBr compounds suffer from the same weaknesses as RbBr:Tl^+^. Their intrinsic X-ray absorption is insufficient and they are very moisture sensitive. These deficits are not shared by the CsBr based phosphors. In view of the reasonable CE and very low SE CsBr:Ga^+^ was proposed as storage phosphor for a NIP in 1999 [75]. The decay-time of the Ga^+^ luminescence is of the order of 20 μs [76]. For this reason and in view of its PSL wavelength of 515 nm, this phosphor must be used in a line scanner [77], in which the phosphor plate is stimulated line by line (Figure 22). In a line scanner efficient read-out is possible with a line scanning time of the order of 1 ms.

**Figure 23:**
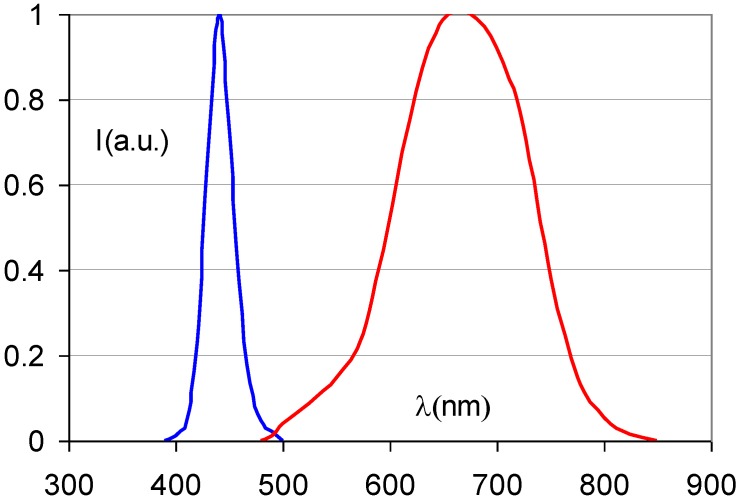
Emission and stimulation spectrum of CsBr:Eu^2+^ (data from [78]).

It soon became clear, however, that Eu^2+^ is the dopant of choice in CsBr [78,79]. Figure 23 shows the emission spectrum, centered at about 440 nm which allows efficient conversion with a photomultiplier tube.

The short Eu^2+^ characteristic decay-time makes fast read-out possible and the material has a convenient stimulation spectrum maximum at 680 nm.

**Figure 24:**
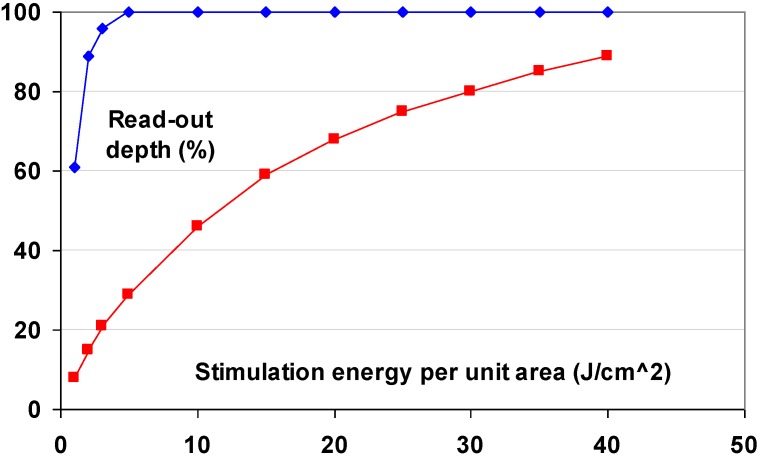
Read-out depth as a function of read-out energy for CsBr:Eu^2+^ and BaFBr:Eu^2+^ (data from [78]).

CsBr:Eu^2+^ has a light output per absorbed dose that is higher than that of BaFX:Eu^2+^. The CE is about 35 pJ/mm^2^/mR *vs*. about 25 pJ/mm^2^/mR for the best BaFX:Eu^2+^ materials. Less light is needed to stimulate the phosphor, allowing the use of a less powerful laser and erasure source in the scanner (Figure 24).

The stimulation energy is only 4 μJ/mm^2^ as for CsBr:Ga^+^. CsBr:Eu^2+^ needle imaging plates have been introduced for general radiography by Agfa in 2005. More recently, NIP’s became available for mammography as well [80].

## 8. The CsBr:Eu^2+^ PSL Mechanism

CsBr has a primitive cubic lattice in which both atoms have eightfold coordinations. The Br^-^ ions are positioned on the lattice points at the edges of the cube, while the Cs^+^ ions fill the cubes’ center. This structure is shared with CsCl and CsI.

Before discussing the CsBr:Eu^2+^ PSL mechanism it is necessary to highlight the effect of annealing. The PSL efficiency can be greatly enhanced by heating vapor deposited CsBr:Eu^2+^ [81] or the phosphor powder [81,82]. Hackenschmied reported that a thermal treatment at 190 °C for a few hours (annealing) increases the PSL output by a factor 9 for CsBr:Eu^2+^ powder [82]. Annealing for longer times or at higher temperatures (overannealing) reduces the PSL output again to almost zero. Similar behavior is observed for vapor deposited CsBr:Eu^2+^ needles plates. Only weak PSL properties are observed after production by thermal vapor deposition and a strong enhancement is realized by annealing in the temperature range of 30–180 °C for a length of time depending on the temperature [81,83]. The annealing atmosphere plays an important role.

**Figure 25:**
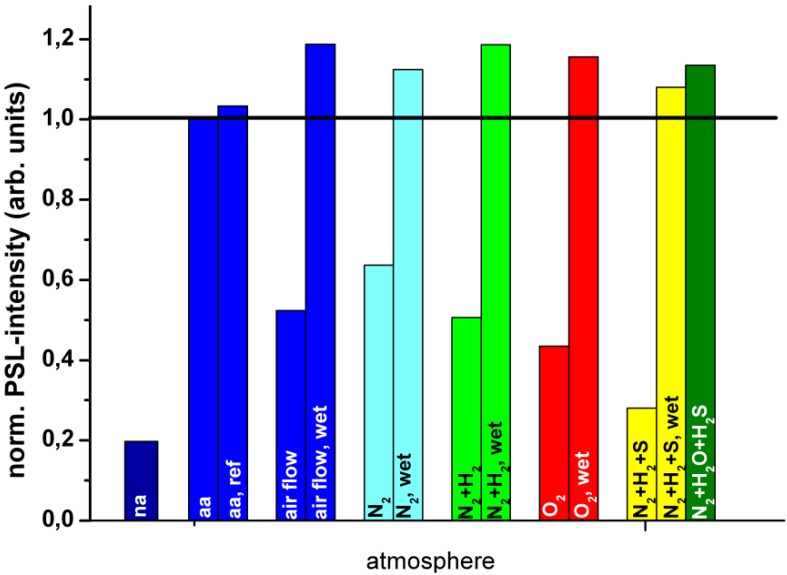
PSL enhancement following annealing at 170 °C for 4 hours in different atmospheres [84] (reproduced with permission from Martina Weidner).

Annealing was performed in different atmospheres at 170 °C for 4 hours [84]. The results, shown in Figure 25, indicate that the PSL build-up is coupled with the presence of humidity. The presence of O_2_ is not sufficient and not even required. In moist air or O_2_, but also in moist N_2_ and N_2_ + H_2_ a stronger PSL increase is achieved than in dry air. Recently, Appleby *et al*. confirmed the essential role of water by demonstrating that the PSL capacity increases in high relative humidity conditions, even at RT [85].

The conformity of the CsBr:Eu^2+^ stimulation spectrum with the CsBr F-center absorption spectrum indicates that the mobile electrons produced in X-ray exposure are trapped in Br^−^ vacancies to form F(Br^−^) centers [86]. The CsBr F-center absorption band of is at 633 nm at 4 K [87]. Taking into account the spectral broadening and red-shift in the transition to RT [87] the stimulation spectrum of CsBr:Eu^2+^ conforms very well to the F-center absorption band.

There is more discussion on the nature of the hole trap and on the involvement of the Eu^2+^ activator and of impurities in the PSL process. Hackenschmied *et al*. prepared CsBr:Eu^2+^ crystals in a single step process having 1 to 5 mol% Eu concentrations. XRD spectra show the formation of additional phases during annealing that were believed to be CsEuBr_3_ and Cs_4_EuBr_6_ [88,89]. The hypothesis was that at least one of these second phases plays a role in hole stabilization. It was suggested that a Eu^2+^-O^−^ center within the precipitates acts as hole trap. A later publication claimed that the foreign phases can be detected in vapor deposited CsBr:Eu^2+^ by means of electron microscopy and that they are present even at Eu-concentrations as low as 50–200 ppm [90]. According to the same study, the presence of the ferroelectric CsEuBr_3_ Perovskite phase makes CsBr:Eu^2+^ measurably ferroelectric [90,91]. It was suggested that the ferromagnetic precipitates stabilize charge separation via their electric field. Thus, exciton formation becomes less likely, which leads to more efficient charge trapping. An alternative explanation given is that the interaction of Eu-Vac_Cs_ centers with the dipole moment of the precipitations would positively influence PSL efficiency by suppressing the formation of PSL-inactive trimers. In later studies, however, the observation of additional phases by electron microscopy could not be repeated [92]. More doubt was cast on the involvement of second phases, when it was demonstrated that, even in ambient conditions, CsEuBr_3_ undergoes a fast temporal phase change to Cs_2_EuBr_5_.10H_2_O which contains Eu^3+^ rather than Eu^2+^. This change makes them PSL inactive [93,94]. It was further demonstrated that the PSL yield is not proportional to the concentration of the segregated compound and that the XRD spectrum of the foreign phases corresponds to the XRD spectrum of Cs_2_EuBr_5_.10H_2_O rather than to the XRD spectrum of CsEuBr_3_ [93].

Weidner studied the luminescence centers in CsBr:Eu^2+^ NIP’s [84]. She demonstrated that different Eu^2+^ based emission centers exist in non-annealed plates. One with a characteristic vibronic low temperature spectrum is produced by annealing and disappears again upon over-annealing [83]. It was concluded, therefore, that this is the only PSL relevant center. During annealing centers are destroyed which exhibit luminescence that is shifted to slightly longer wavelengths but are not PSL-active. Annealing transforms them into the characteristic “vibronic” centers. The build-up of the luminescence of the “vibronic” centers is correlated with the build-up of PSL activity during annealing in humid atmosphere. Upon overannealing the “vibronic” centers are destroyed and transformed into a third type of center with a slightly higher energy photoluminescence emission and which is not PSL active. No information is presented, however, on the nature of the different centers.

The study further demonstrates that the number of PSL-active traps is reduced by overannealing. After over-annealing, more direct recombination of charge carriers occurs upon X-ray exposure and more charges are stored in parasitic traps in which trapping is metastable at RT. The overall conclusion is that overannealing leads to destruction of the PSL-active luminescence centers and that the formation of PSL relevant trapping centers is less effective. The correlation between humidity during annealing and PSL build-up leads Weidner to the hypothesis that Eu-H_2_O or Eu-hydrogen aggregates could be the origin of the vibronic emission. An alternative suggestion is that aggregates of multiple Eu-centers are formed, whereby water or hydrogen promotes the diffusion of single Eu^2+^ ions.

**Figure 26:**
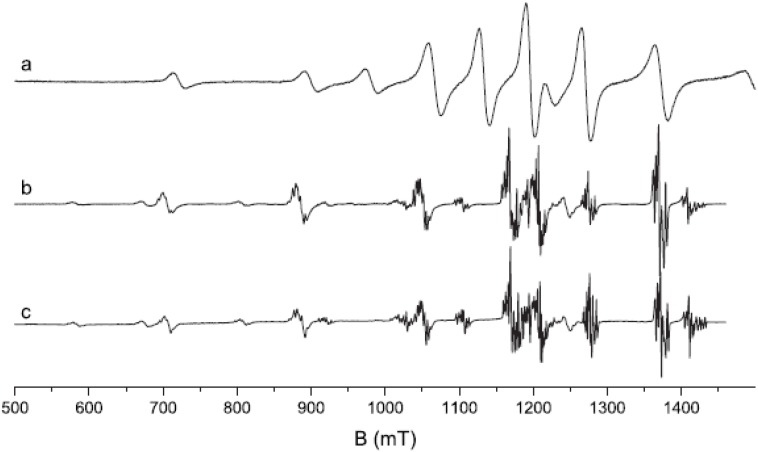
Normalized Q band spectra with magnetic field ⊥ to the plate (<100> spectrum) of an annealed Agfa NIP at RT (**a**) and at 4 K (**b**) and of a commercial NIP at 4 K (**c)** [98] (reproduced with permission from Frank Loncke).

**Figure 27:**
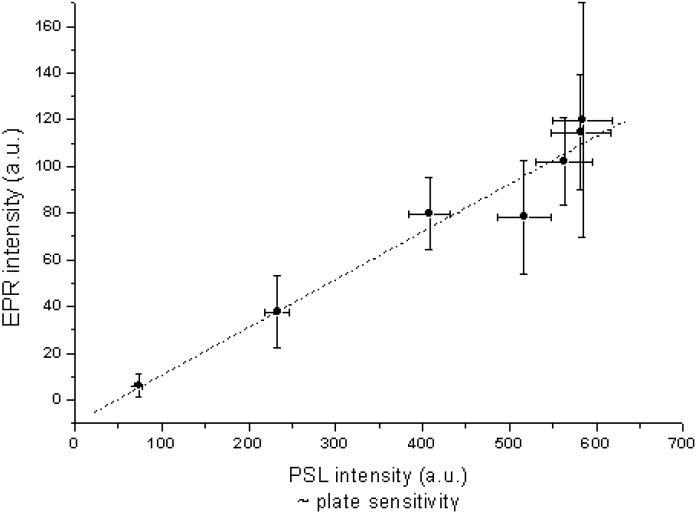
Intensity of the characteristic EPR signal *vs*. PSL activity for samples of the same NIP treated in different ways [98] (reproduced with permission from Frank Loncke).

Loncke *et al*. studied the nature of the luminescence center(s) involved in CsBr:Eu^2+^ PSL by EPR and ENDOR [95,96,97,98,99]. Models are presented for the Eu based defects, in the different annealing stages that explain many features. Non-annealed, annealed and over-annealed vapor deposited CsBr:Eu^2+^ were studied. Different EPR signals were observed in different stages of the annealing cycle. For samples annealed in the region between RT and 300°C a signal (Figure 26a) was observed with an intensity that increased proportionally to the PSL activity (Figure 27). This signal was assumed to be coupled, therefore, with a defect involved in energy storage and release.

**Figure 28:**
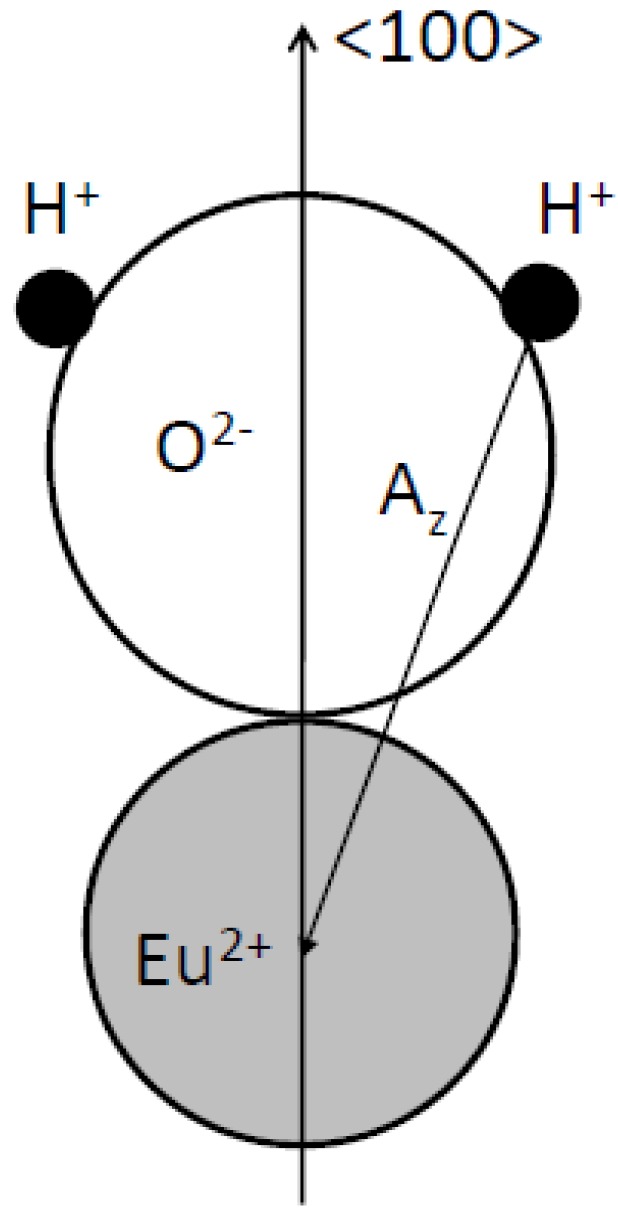
Model for the Eu^2+^-H_2_O couple in CsBr:Eu^2+^, consistent with the EPR spectrum of annealed CsBr:Eu^2+^ NIP’s [98] (reproduced with permission from Frank Loncke).

The signal could not be obtained in CsBr:Eu^2+^ single crystals. Based on the observation that the room temperature EPR spectrum of annealed CsBr:Eu^2+^ needle plates is due to a single paramagnetic center with S = 7/2 which exhibits a large zero field splitting it is concluded that the defect is Eu^2+^ centered [96]. The defect has its main tetragonal axis along a crystal <100> direction. The EPR spectrum made at 4 K (Figure 27b) can be explained by the presence of 2 defects, one with tetragonal and one with nearly extreme rhombic symmetry [97]. The LT spectrum undergoes drastic changes in the 30–40 K range. It gradually evolves into the RT spectrum dominated by one type of tetragonal Eu^2+^ center beyond 40 K. For both defects, all principal axes are again oriented along <100> directions. A thorough analysis of the EPR spectrum demonstrates that in the 2 defects, Eu^2+^ is associated with a H-containing impurity. In both centers the Eu^2+^-H interconnection line is found close to a <100> direction. Moreover, the ^1^H HF coupling strength suggests that the proton belongs to a H_2_O ligand of the Eu^2+^ ion (Figure 28).

Expressing the ZFS parameters for the three EPR type defects (including the RT variant) in a common axis system, taking the Eu-OH_2_ axis as the z axis, reveals striking similarities. It is, therefore, assumed that they have a common core, differing only in the position of an additional perturbing lattice defect. The Eu^2+^ ion and H_2_O molecule are believed to (approximately) occupy nearest-neighbor Cs^+^ positions and to exhibit an important relaxation toward one another.

**Figure 29:**
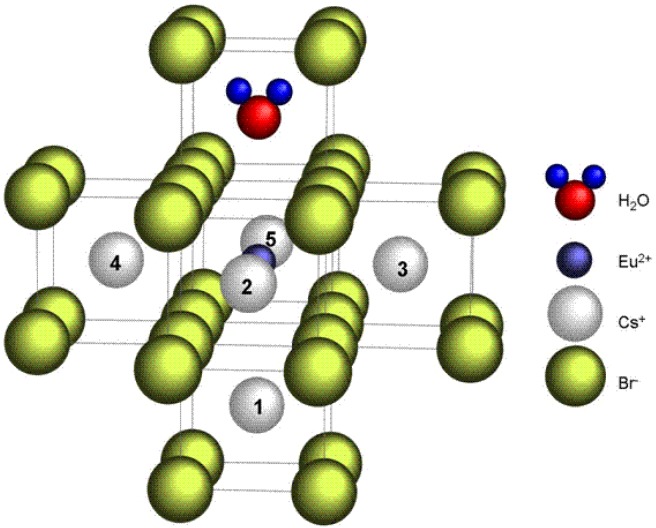
Possible models for the luminescent centers in annealed CsBr:Eu^2+^ [98] (reproduced with permission from Frank Loncke).

The perturbing defect is proposed to be a cation vacancy in one of the five remaining nearest-neighbor positions to the Eu^2+^ ion (Figure 29). The position of the vacancy, either along the Eu-OH_2_ axis or in one of the four equivalent positions in the plane perpendicular to it, explains the difference between the 2 EPR spectra at low temperature. The disappearance of the rhombic defect at higher temperatures can be explained by hopping of the vacancy between these positions at elevated temperatures which produces the axial EPR spectrum [98,99]. Quantum chemical calculations are necessary in order to verify the stability of these models for the defects present in CsBr:Eu^2+^ after annealing and which apparently play a key role in the PSL process in CsBr:Eu^2+^ NIP’s, as well as for revealing their formation mechanisms [99]. The attraction between Eu^2+^ and the O atom of the water molecule can stabilize the Eu^2+^ ion and prevent diffusion and aggregation. The model has one negative charge in excess. The vacancy is not required for charge compensation, but may accommodate local stress induced by the impurity substitution. It may also attract a Br^−^ vacancy that can form an F-center after X-ray exposure.

At about 300 °C the discussed EPR signal disappears and a broad new signal appears at g ≈ 2. It is believed that at this temperature the stabilizing defects diffuse away, so that the Eu^2+^ ions can form micro phases as e.g., Cs_x_Eu_y_Br_z_. At 500 °C a new EPR signal was detected that is short-lived. It could only be measured immediately after overannealing or when the sample was quenched in liquid N_2_. These signals are believed to originate from unstable Eu^2+^ monomers. Overannealing leads to incorporation of the Eu dopant in a solid-state matrix in a way similar to that of the melt-grown CsBr single crystals. These solid-state matrices do not give rise to an EPR signal.

The above models for the Eu^2+^ centers are not strictly proven, but are very plausible and on the basis of the available information no better models can be given. Careful IR analysis might, in principle, help discriminate between OH^−^ and H_2_O as neighbors to Eu^2+^ in the annealed CsBr:Eu^2+^ lattice. However, CsBr:Eu^2+^ very rapidly attracts surface water that is very difficult to remove, even by heating in vacuum. The surface water IR signal hides the signal of OH^−^ and/or H_2_O impurities incorporated in the CsBr crystal. It must also be stressed that it is very unlikely that OH^−^ can substitute for Cs^+^ in the CsBr lattice. OH^−^ would probably replace Br^−^, but this substitution would lead to <111> as main symmetry axis, possibly further lowered by additional perturbations and then leading to monoclinic or triclinic symmetry.

It is known that incorporation of Eu^2+^ in alkali halides leads to neighboring cation vacancies [100]. However, based on the assumption that the presence of O_2_ during annealing is essential for the PSL enhancement [85], Hesse *et al*. suggested that not Eu^2+^-V_Cs_ dipoles are involved in the PSL process, but that oxygen must play a role [94]. Later it was proposed that the hole trap is a [Eu^2+^-O^2−^] dipole. In a subsequent study, CsBr:Eu^2+^ was annealed in an H_2_S atmosphere and it was shown that this produced at least the same PSL increase as annealing in O_2_ atmosphere [93]. It was claimed that [Eu^2+^-S^2−^] dumbbells are created as PSL active sites. Measurements indicated a slightly reduced PSL lifetime for the sulfur treated materials compared to the oxygen treated ones indicating a spatial correlation between the sulfur ion and the Eu^2+^ ion. The dumbbells or dipoles exhibit strong dipole moments and are claimed to be responsible for better charge separation of the electron/hole pairs after X-ray exposure. The improved PSL activity after annealing is explained by the fact that annealing increases the number of [Eu^2+^-O^2−^] (or [Eu^2+^-S^2−^]) dipoles. O^2−^ (or S^2−^) also charge compensates for Eu^2+^ in the CsBr lattice. When it was later realized that the presence of water, and not oxygen, is essential in the annealing process, two different explanations for its role were given. One is that H_2_O may be an even better source for the creation of [Eu^2+^-O^2−^] dipoles than O_2_ as a result of the following reactions during annealing [85]:

(6) $$\begin{array}{l} 2\;\text{CsBr}+H_{2}O\overset{\;}{\to }{\text{Cs}}_{2}O+2HBr\\ EuBr_{2}+Cs_{2}O\overset{\;}{\to }\text{EuO}+\text{2CsBr} \end{array}$$

In addition, the hypothesis was presented that water is incorporated in the CsBr lattice, to the extent that up to one molecule of H_2_O is absorbed per unit cell [85]. The H_2_O molecules are oriented by the [Eu^2+^-O^2−^] dipoles. This enhances the electric field around the dipoles, and, as a consequence, the charge trapping efficiency.

**Figure 30:**
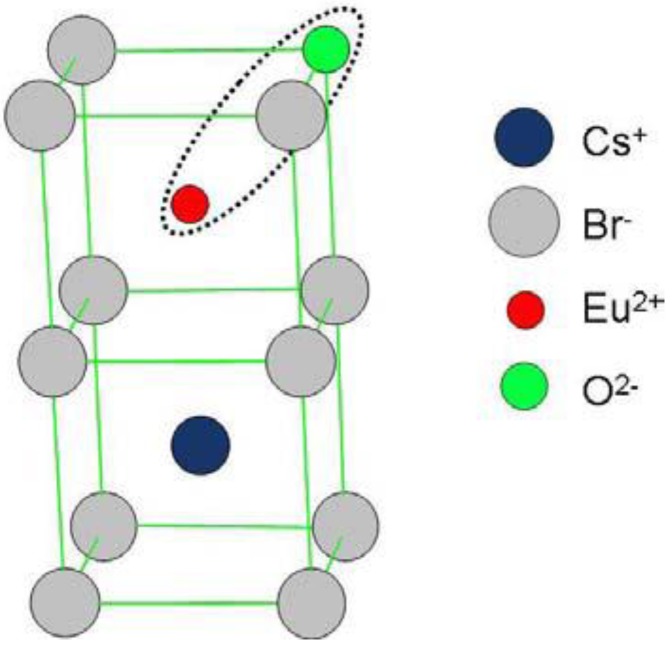
[Eu^2+^-O^2−^] dipole orientation in the CsBr lattice [98] (reproduced with permission from Frank Loncke).

The fact that H_2_S annealing has a similar effect as annealing in (humid) air and that S is incorporated in the vicinity of Eu^2+^ as asserted by Hesse *et al*. [93], is also consistent with the luminescent center model, proposed by Loncke *et al*. [98], in which H_2_S may replace H_2_O. It must be remarked that H_2_S is rather an analogue of H_2_O than of O_2_. The [Eu^2+^-O^2−^] dipoles proposed by Hesse *et al*. [93] have both ions placed on neighboring cation/anion positions (Figure 30). This results in a <111> oriented dipole. The orientation of the defect center coupled with the CsBr:Eu^2+^ PSL activity according to the study by Loncke [97] is oriented along the <100> direction. The [Eu^2+^-O^2−^] dipole model is not consistent with the annealed CsBr:Eu^2+^ EPR and ENDOR results.

## 9. Image Quality of CR Systems

In order explain image quality in terms of phosphor plate quality a mathematical model is derived containing parameters that can be correlated with phosphor plate physical characteristics. It should be pointed out, however, that the ultimate test of image quality in medical imaging is diagnostic accuracy. Therefore, reference is made to results of clinical studies in Paragraph 9.4.

### 9.1. Definition of Medical Radiography System’s Image Quality

#### 9.1.1. Signal-to-Noise Ratio (SNR)

The X-ray quanta used as image carriers in radiography can be considered as independent random events in time and space. Therefore, the number of quanta per unit area and time fluctuates according to Poisson statistics. When making a flat-field image (without any object on the detector) the number of quanta incident per unit area has a mean (signal), and a variance (noise). Poisson statistics allows calculation of SNR based on the photon fluence. The mean number of incident quanta equals the fluence, while the variance is the square root of the fluence. Hence, SNR^2^ is equal to the fluence or the average number of quanta incident on the detector. This is the highest value of image SNR^2^ that can be reached with this number of quanta by any X-ray imaging system.

#### 9.1.2. Detective Quantum Efficiency (DQE)

Since the incoming SNR is proportional to the square root of the dose and since the dose to which a patient is exposed should be as low as possible, the best radiography system is the one that degrades incoming SNR the least, *i.e*., the system that has the highest SNR_out_ for a given SNR_in_. In this sense, detective quantum efficiency (DQE), defined as:

(7) $$DQE=SNR_{out}^{2}/SNR_{in}^{2}$$

Expresses how effectively a system captures the information content of an X-ray image. DQE is between 0 and 100% and is widely used in scientific, regulatory and commercial communities as a fundamental measure of an X-ray imaging system’s performance.

#### 9.1.3. Noise Sources

CR systems, as all radiography systems, have a number of noise sources that degrade DQE. Only incoming photons that interact with the active layer of the detector produce signal and contribute to the noise. If the storage phosphor layer’s absorption equals α, SNR^2^ is reduced to αQ, where Q is the quantum fluence. In medical radiography this is often the most important reason for image quality degradation. CR plates can have absorptions as low as 25–30% depending on plate type and X-ray spectrum. By this, the DQE is reduced to maximum 25% to 30%.

Apart from quantum noise, resulting from the finite number of quanta per unit area interacting with the plate, there are a number of other noise sources.

Electronic noise is produced by the electronic components in an imaging system. Examples in CR are fluctuation of the scanning laser output and fluctuation of signal amplification in the PMT. The IP does not contribute to this type of noise and electronic noise in a CR system should be at a very low level and should not play a role at the medical doses. It has been illustrated, however, that in the line scan digitizer with CCD detector, DX-S^®^, a minor contribution of electronic noise is present. For flying spot digitizers, it can further be disregarded.

Secondary quantum noise or amplification noise is related to the conversion process from X-ray quanta to photo-electrons in the PMT or CCD of the scanner. The light photons produced in the PSL process can also be considered as random events, following Poisson statistics and will add noise. The higher the gain of the CR system, *i.e*., the higher the number of photo-electrons in the scanner detector per absorbed X-ray quantum in the CR plate, the less secondary quantum noise will be prominent. System gain must be higher than 5 to limit the impact of secondary quantum noise. Even then it will affect small detail visibility.

So-called Poisson excess noise is the collection of a number of noise contributions. One origin is the fact that different layers in the detector have different contributions to the final image. Due to the turbid character of the IP, the intensity of the stimulation light diminishes with the depth in the phosphor layer. In addition, emission photons from the deeper layer have lower probability to reach the detector. A more transparent phosphor layer will lead to a smaller contribution of this noise type. Poisson excess noise has also an X-ray component. The X-ray spectrum is not monochromatic and the K-photons produced in the interaction with the phosphor crystals have a certain probability of escape from the IP, also depending on the location of the interaction in the phosphor layer. As a result, the energy deposited by an absorbed X-ray quantum varies, which again adds noise.

Fixed pattern noise or screen-structure noise is very much related to IP quality. The IP light scattering behavior varies slightly from pixel to pixel due to internal structures in the phosphor layer and in substrate and top-coat. As a result, the direction in which the laser beam is deflected in the scanning process varies slightly as well as the size of the halo produced in the phosphor layer. It is clear that substrate and top-coat should have as little structure as possible in order to keep screen-structure noise minimal. Since screen-structure noise is proportional to the X-ray dose whereas quantum noise is proportional to the square-root of the dose, screen-structure noise increases in importance with increasing dose level. Therefore, it has a larger contribution in mammography where higher doses are used than in general radiography.

#### 9.1.4. Noise as a Function of Spatial Frequency

Variance is a poor measure of noise in a medical image. It does not account for the spatial distribution of the noise.

**Figure 31:**
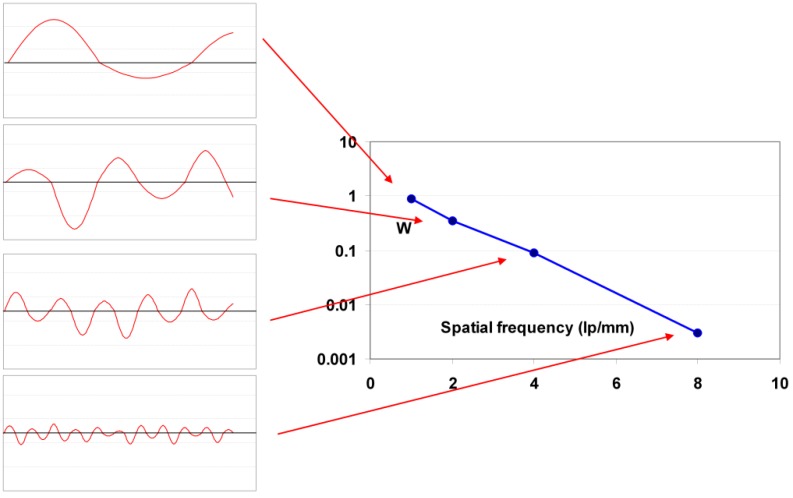
Schematic representation of Wiener spectrum concept.

Different noise types have different spatial distributions. Quantum noise is white noise, meaning that the noise power is identical for all spatial frequencies. Screen-structure noise, on the other hand, can have almost any distribution sometimes with maxima at specific spatial frequencies related to periodic structures in the IP originating from the coating process. For that reason, it is important to use the noise power spectrum or Wiener spectrum (W) that represents the variance of the signal, but distributed as a function of spatial frequency, ν. Noise in an image has contributions of all spatial frequencies and the Wiener spectrum, W, gives the amplitude of each of these contributions (Figure 31). It shows at which frequencies variations are strong and at which frequencies variations are weak. Spatial frequency is a measure of how often sinusoidal components (obtained by Fourier transform) of an image signal repeat per unit of distance. A unit of spatial frequency is cycles per millimeter. In radiography, the unit of line pairs per millimeter (l p/mm) is incorrectly used, where cycles per millimeter is meant (strictly, l p/mm relates to a square wave and not to a sinusoidal wave).

#### 9.1.5. Resolution and Modulation Transfer Function (MTF)

**Figure 32:**
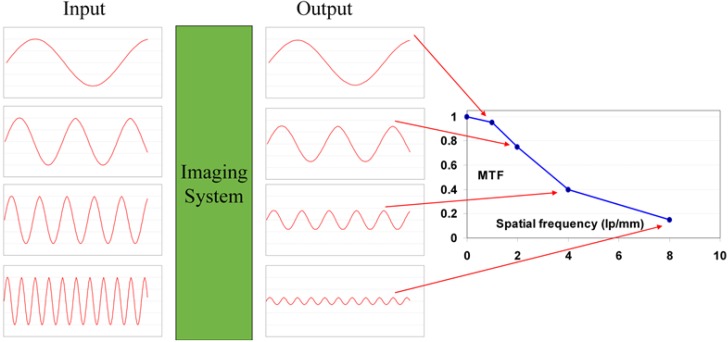
Schematic representation of the MTF concept.

The function of a radiography system is to translate the modulations of the X-ray beam transmitted by the patient into a visible image and to reproduce them faithfully to clearly visualize the details of body parts. However, any radiography system not only degrades image quality by introducing extra noise. It also reduces the modulations, thereby blurring the image. The laser beam used to scan the image in medical CR, generally, has a diameter between 50 and 100 μ. In the phosphor layer a halo is produced which may become several hundred micrometers wide. It is clear that this will cause a significant reduction in signal height for image details smaller than the laser halo in the phosphor layer. The reduction of the modulation is expressed by the modulation transfer system (MTF). The MTF is the ratio of the modulation of a sinusoidal signal leaving to that entering an imaging device and decreases with increasing spatial frequency (Figure 32).

Unsharpness will reduce both the useful signal amplitude as the noise amplitude. The signal amplitude will be more affected, however, because the signal is blurred in every step of the imaging chain, while noise introduced in a certain step is blurred only by the subsequent steps and not by the previous ones. Hence, only X-ray quantum noise is reduced to the same extent as the signal by the system MTF, because it is present from the first stage in the imaging chain. All other noise components are reduced to a lesser extent. Therefore, unsharpness degrades the DQE, especially at higher spatial frequencies.

#### 9.1.6. DQE as a Function of Spatial Frequency

Since signal and noise vary as a function of spatial frequency, DQE is spatial frequency, ν, dependant:

(8) $$DQE\left(\nu \right)=SNR{\left(\nu \right)}_{out}^{2}/SNR{\left(\nu \right)}_{in}^{2}$$

A CR system is not isotropic and leads to differences in noise and MTF between fast- and slow-scan directions. This makes DQE vary differently in these 2 fundament directions. It must be expressed as a functional of the spatial frequencies in those 2 directions, *u* and *v*:

(9) $$DQE\left(u,\nu \right)=SNR{\left(u,\nu \right)}_{out}^{2}/SNR{\left(u,\nu \right)}_{in}^{2}$$

The signal produced by the CR system is reduced by the MTF. Replacing SNR(u,ν)_in_^2^ by Q, this leads to:

(10) $$DQE\left(u,\nu \right)=\frac{S^{2}\cdot MTF{\left(u,\nu \right)}^{2}}{Q\cdot W_{out}\left(u,\nu \right)}$$

A CR system is a linear detector with zero intercept. Standard procedure for such a system is to determine the relation between the output signal and the exposure in number of quanta per unit area:

(11) S=c⋅Q

This relationship gives the following DQE equation:

(12) $$DQE\left(u,\nu \right)=\frac{c^{2}\cdot Q\cdot MTF{\left(u,\nu \right)}^{2}}{W_{out}\left(u,\nu \right)}$$

The IEC standard expression is:

(13) $$DQE=\frac{Q\cdot MTF{\left(u,\nu \right)}^{2}}{W_{out}\left(u,\nu \right)}$$

where it must be understood that W is obtained from images that have been linearized to quanta per unit area at detector input.

As a conclusion, DQE measurement implies measurement of MTF(u,ν) and noise power spectrum, W_out_(u,ν), and of the number of X-ray quanta incident on the detector per unit area, Q.

In practice, the following relation is used to determine the quantum fluency:

(14) $$Q=K_{a}\cdot SNR_{in}^{2}$$

where K_a_ is the air kerma, measured with an ionization chamber and the SNR^2^ values are tabulated for well-defined exposure conditions [101].

### 9.2. Measurement of Image Quality

#### 9.2.1. Measurement of MTF

Since MTF represents the reduction of the modulation of a sinusoidal signal by the imaging system, the easiest way to measure it is to expose the detector trough a phantom with sinusoidal absorption variation. Manufacturing of such a phantom is, however, not practical. The standard way of measuring MTF is, therefore, by analyzing an edge image. 

**Figure 33:**
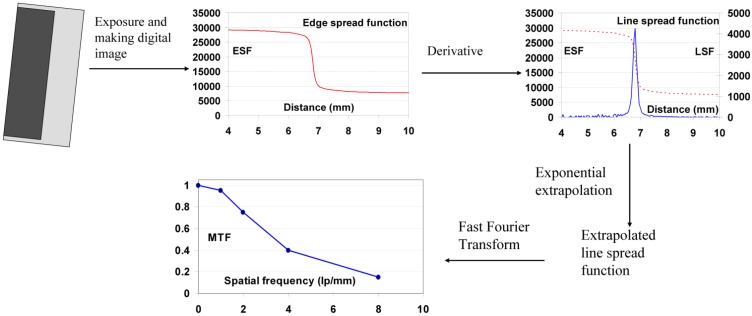
Schematic presentation of MTF measurement with edge phantom.

All lines of the edge image, perpendicular to the edge are scanned and averaged with leveling due to zero intensity. Thus, the edge spread function or edge profile function is obtained. The distances in pixels are corrected depending on the angle of edge slope. The edge should be sloped for smooth averaging. Next the first derivative from edge spread function is taken. The result is the line spread function. By applying discrete Fourier transform to the line spread function the MTF is obtained [102]. The procedure is schematically shown in Figure 33.

#### 9.2.2. Measurement of W

For the measurement of W several homogeneously exposed images or flat-field images are recorded. The images are converted to obtain pixel values proportional to the exposed number of quanta (9.1.6). A 2-dimensional 2nd order polynomial is fitted to the image and this function is used for de-trending of the low frequency profile due to exposure inhomogeneity (Heel effect). The central part of the image is divided in overlapping areas of 256 pixels by 256 pixels wide and in each sub-image the Wiener spectrum is calculated [101,103,104]. W(u,ν) is obtained by averaging the samples of all the spectra of the sub-images. A minimum number of independent pixels must be used to guarantee good experimental accuracy. The conversion of the 2D Wiener spectrum to 1D Wiener spectra in both fundamental orientations is in the IEC62220-1 [101] defined in a region of 14 rows/columns around both axes. 

#### 9.2.3. Measurement of Air Kerma and Translation to Number of Quanta

Kerma originates from the acronym, KERMA, for Kinetic Energy Released per unit Mass (of air). Air Kerma is a measure of the amount of radiation energy, in Joules (J), absorbed in a unit mass (kg) of air. Therefore, it is expressed in the units of J/kg or Gray (G). It is measured with an ionization chamber. Since the ionization produced in air by radiation is proportional to the energy released, ionization chambers can be used to measure exposure: a conversion factor, ϕ, can be used to convert air kerma to exposure values. The conversion factor can be obtained from X-ray spectral modelling [101]:

(15) $$\phi =\frac{\int n(E)\cdot f(E)\cdot dE}{\int n(E)\cdot dE}$$

where n(E) is the relative number of quanta in an X-ray spectrum of energy E and f(E) is the number of quanta per area per Air Kerma at energy E. Since different models can lead to different results, the IEC standard defined the conversion factor. An “official” DQE is measured under standard conditions, whereby use is made of the defined conversion factor. For example, a standard general radiography exposure condition is RQA 5, for which a general radiography source is used with 21 mm thick external Al filtering. The X-ray spectrum should have an Al half-value layer of 7.1 mm and leads to a conversion factor of 30.17 quanta/(mm^2^.nGy). 

### 9.3. Relation between Imaging Plate Characteristics and Image Quality (DQE)

#### 9.3.1. Simple DQE Model

A simplified model is derived that allows qualitative explanation of the relation between DQE and the physical characteristics of the CR IP.

As explained, quantum noise in CR images is partly X-ray quantum noise and partly amplification noise. An IP with absorption α, leads to an amount of X-ray quantum noise equal to:

(16) $$N_{Q}=\sqrt{\alpha \cdot Q}$$

This noise contribution is reduced by the CR system MTF and amplified with the gain of the system. Quantum noise is multiplied by the “Poisson excess” factor, (1 + β)^1/2^ [105,106], which represents the effect of the depth-dependence of the gain factor. According to this approach quantum noise on signal level is equal to:

(17) $$N_{Q}\left(\nu \right)=\sqrt{\left(1+\beta \right)\cdot \alpha \cdot Q}\cdot G\cdot MTF\left(\nu \right)$$

Amplification noise, due to fluctuation of the number of photo-electron per absorbed X-ray quantum, is, based on Poisson statistics, equal to:

(18) $$N_{A}\left(\nu \right)=\sqrt{\alpha \cdot Q\cdot G}$$

Since screen-structure noise can, in principle, have any frequency dependence, and since its contribution is proportional to the dose, a general expression for this noise is:

(19) $$N_{SS}{\left(\nu \right)}^{2}=Q^{2}\cdot W_{ss}\left(\nu \right)$$

Based on Equations (17–19), total image noise power is:

(20) $${N_{T}}^{2}=N_{Q}^{2}+N_{A}^{2}+N_{ss}^{2}=\alpha \cdot Q\cdot G\cdot [\left(1+\beta \right)\cdot G\cdot MTF{\left(\nu \right)}^{2}+1]+Q^{2}\cdot W_{ss}\left(\nu \right)$$

This leads to the following expression for SNR_out_^2^:

(21) $$SNR_{out}^{2}=\frac{\alpha \cdot Q\cdot MTF{\left(\nu \right)}^{2}}{\left(1+\beta \right)\cdot G\cdot MTF{\left(\nu \right)}^{2}+1+\frac{Q\cdot W_{ss}\left(\nu \right)}{\alpha }}$$

Or

(22) $$SNR_{out}^{2}=\frac{\alpha \cdot Q}{1+\beta +\frac{1}{G\cdot MTF{\left(\nu \right)}^{2}}+\frac{Q\cdot W_{ss}\left(\nu \right)}{G\cdot MTF{\left(\nu \right)}^{2}}}$$

SNR_in_^2^ being Q, the following DQE formula is obtained:

(23) $$DQE=\frac{\alpha }{1+\beta +\frac{1}{G\cdot MTF{\left(\nu \right)}^{2}}+\frac{Q\cdot W_{ss}\left(\nu \right)}{\alpha \cdot G\cdot MTF{\left(\nu \right)}^{2}}}$$

#### 9.3.2. Relation between DQE and IP Characteristics

DQE is proportional to X-ray absorption, α (Equation (23)). In commercial CR IP’s high absorption is achieved by using high density materials having a K-edge at suitable X-ray energy. The use of high phosphor layer thickness or coating weight also contributes. There are limitations to the useful thickness, however. The intensity of the laser light used to scan the IP diminishes with depth. It does not penetrate indefinitely in the phosphor layer. A powder phosphor layer scatters the light isotropically and has a maximum useful thickness of 250 to 300 μ. F-centers produced in deeper layers are not reached by stimulation light and light emitted by deeper layers would not reach the scanner light detector. For general radiography, this limits the useful X-ray absorption to about 30%. In the NIP phosphor layer stimulation and emission light is preferentially scattered in forward direction. Light travels through the phosphor layer over much larger distances in this direction. As a result, the Genrad NIP phosphor layer can be twice as thick as the PIP phosphor layer. In addition, the needles are grown on a substrate without the need for a binder. The phosphor volume content of the active layer is about 90% *vs*. only about 70% in a powder screen. Both factors together allow the use of Genrad NIP’s with more than twice the X-ray absorption of Genrad PIP’s. Equation (23) demonstrates that this, in itself, is sufficient to double DQE.

Screen-structure noise is the only noise component in Equation (23) that increases with increasing dose. In the general radiography dose range its impact on DQE is small if a decent IP is used. For the higher doses used in mammography screen-structure noise has a measurable impact on DQE. In many NDT applications screen-structure noise is the most important noise source. Exposures are used for which SNR saturates. The saturation level is determined by the degree of screen-structure noise (Equation (22)). For PIP, screen-structure noise, generally, diminishes with decreasing phosphor particle size. The quality of the lacquer coating and drying processes obviously are important as well. The NIP PVD production process leads to more homogeneous layers than the powder phosphor coating process. As a result, NIP images have less screen-structure noise, which is a more important benefit for mammography.

When screen-structure noise is disregarded, a simplified DQE equation results:

(24) $$DQE=\frac{\alpha }{1+\beta +\frac{1}{G\cdot MTF{\left(\nu \right)}^{2}}}$$

To assess the impact of gain, G and MTF, the product G.MTF^2^ in Equation (24) should be as high as possible in order to maximize DQE. However, modifications in IP build-up that improve MTF generally reduce G and *vice versa*. They can be used to tune the IP for a specific application but hardly affect the overall quality level. To clearly visualize large details with small contrast it is better to have a higher gain. By this, DQE will be higher at low spatial frequencies, but it will suffer at high spatial frequencies, because MTF will be reduced. For better visibility of small details it is best to go for a higher MTF at the cost of G. The lower gain will lead to more back-ground noise.

Storage phosphors continue to emit light for a short time after stimulation is stopped. The luminescence decay time depends on the phosphor activator. The light collected from the current position of the laser is mixed with the remaining emission from previous laser positions. This causes a loss of resolution in the fast-scan direction when the laser dwell time per pixel approaches the luminescence decay time. Since both commercial phosphors, BaFX:Eu^2+^ and CsBr:Eu^2+^, have Eu^2+^ as activator, the influence of this phenomenon depends on the read-out pixel time and not on the quality or type of storage phosphor. The main factor limiting spatial resolution in CR is scattering of the stimulation light in the turbid phosphor layer.

1. The diameter of the laser beam halo increases with the depth in the layer. Hence, spatial resolution and MTF in CR decrease with increasing phosphor layer thickness.
2. Another factor of influence is the color of the substrate on which the phosphor layer is coated. The more the substrate absorbs the stimulation light, the higher MTF will be, because reflected stimulation light will diffuse further in the lateral direction, thereby increasing the size of the stimulating halo.
3. Resolution can be improved by mixing anti-halo dye or pigment in the phosphor layer or top-coat. This, obviously, limits lateral light diffusion.
4. The shorter the distance between two light scattering events, *i.e*., the shorter the mean free path of the photons in the phosphor layer, the shorter will be the distance over which light can travel before being absorbed. This explains why the resolution offered by PIP improves with decreasing phosphor particle size and in NIP with decreasing needle diameter.

All described parameters have an impact on sharpness (MTF) in a similar way in PIP and NIP. Since the needles tend to keep the stimulation light inside, travelling parallel to their axis the diameter of the laser beam halo in the phosphor layer is strongly reduced. For equal phosphor layer thickness, the halo will be much smaller in NIP than in PIP. As indicated, phosphor layer modifications that improve MTF always have a negative effect on system gain, G. A reduction of the laser beam halo by using a thinner phosphor layer, by using an absorbing substrate, by using anti-halo dye in phosphor layer or top-coat or by using finer phosphor particles or thinner needles leads to stimulation of less F-centers and less PSL emission and, therefore, to a lower number of photo-electrons in the scanner PMT per absorbed X-ray quantum. The higher transparency of the needle layer leads to a higher escape efficiency of the stimulated emission in NIP, and, as a consequence, to a higher gain in the same scanner. General radiography NIP’s have been combined with a line-scanner (Agfa, DX-S^®^) with a relatively low light collection efficiency. This compensates for the higher G.MTF^2^ product to which NIP’s might give rise. Hence, in a line-scanner, DQE for NIP is not further improved beyond the factor of 2, resulting from the higher X-ray absorption, above PIP + flying-spot scanner level. Since 2010 a flying-spot scanner for NIP is available (Agfa, DX-G^®^). In this scanner DQE for NIP is further improved thanks to a higher G.MTF^2^ product.

The higher transparency of NIP leads to a more homogeneous contribution to the image of the different IP layers and, thus, to a lower value of the Poisson excess factor, β, in Equation (23).

### 9.4. Technical and Clinical Image Quality of CR Systems with PIP and NIP

**Figure 34:**
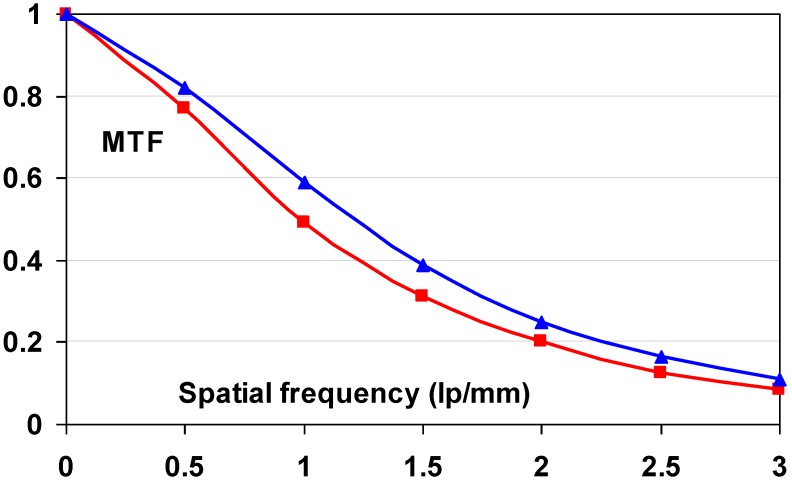
MTF as a function of spatial frequency for a commercial MD-30 PIP, scanned in a commercial scanner (red squares) and for a 500 μ CsBr:Eu^2+^ NIP, scanned in a laboratory scanner (blue triangles) (data from [79]).

When CsBr based NIP’s were first presented [75,78], a prediction was made of the achievable DQE for general radiography, based on the simple Hillen model [107] and the considerations outlined in 9.3.2. The conclusion was that the use of NIP would lead to a significant improvement of CR performance. In a subsequent study the DQE was measured under general radiography exposure conditions for a CR system consisting of a laboratory flying-spot scanner and a 500 μ thick prototype general radiography NIP [79]. The prototype NIP gave a slightly higher MTF than a commercial general radiography PIP in the same scanner (Figure 34).

**Figure 35:**
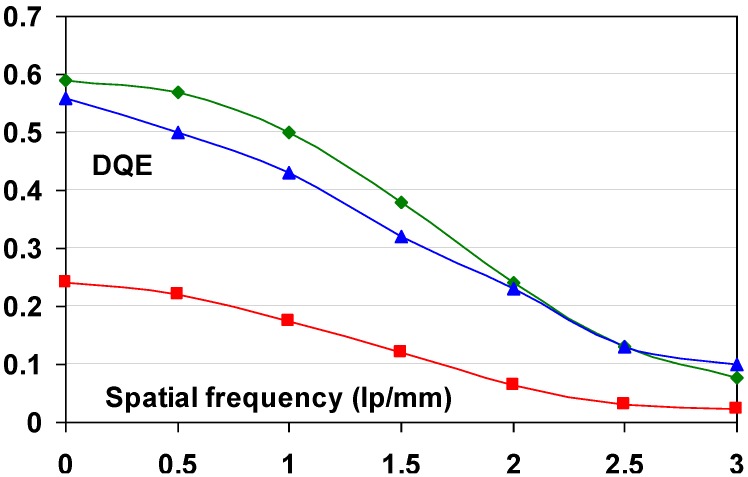
DQE as a function of spatial frequency for state-of-the art CR with PIP (red squares), for a 500 μ CsBr:Eu^2+^ NIP, scanned in a laboratory scanner (blue triangles) and for DR with CsI:Tl (green diamonds) (data from [79]).

Thanks to the much higher X-ray absorption, however, NIP improved the DQE by a factor of 2 at low spatial frequencies and by up to a factor 5 for the higher spatial frequencies (>3 lp/mm) with respect to PIP (Figure 35). It was confirmed in this study that, for general radiography, CR with NIP would be able to match the technical image quality obtained with DR based on CsI:Tl^+^ needle plates and amorphous Si photo detectors.

The results obtained by Shaetzing *et al*. [108] on an experimental line-scanner, the so-called scan-head system, confirmed this conclusion. A similar development and investigation by Fuji, in which a CsBr:Eu^2+^ NIP in a line-scanner was compared to PIP in a flying-spot scanner lead to the same result [109].

**Figure 36:**
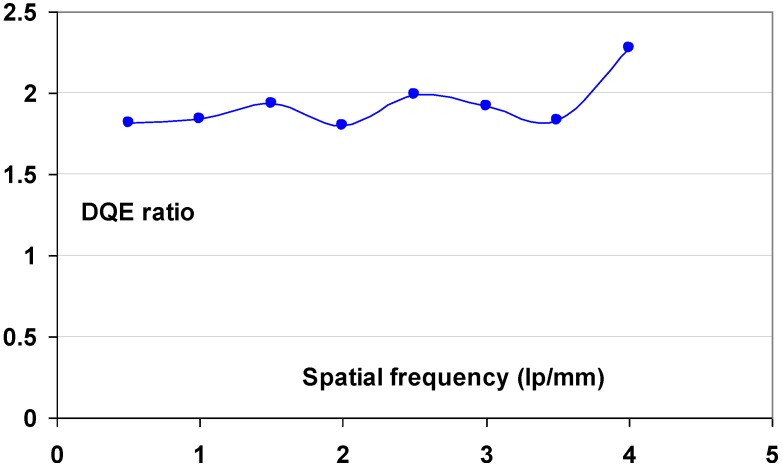
Ratio of NIP system-to-PIP system DQE (data from [93]).

The first commercial CsBr:Eu^2+^ NIP system, DX-S^®^, was introduced by Agfa in 2005. It is a general radiography system in which the plate is scanned with a line-scanner of the scan-head type (Figure 22). Willems *et al*. demonstrated that the system does not make use of the full potential of NIP [110]. In DX-S^®^ the light emitted by the IP is guided to a CCD array for conversion in electrical signals. The CCD detector has a high conversion efficiency of the order of 65%. However, the numerical aperture of the lens system that projects the light emitted by the IP on the CCD is very low. It is less than 10%, which is much lower than the transmission of the light guides used in flying-spot scanners. This has a negative impact on the system gain, G, and on the DQE especially at higher spatial frequencies (Equation 24). Nevertheless, the DQE of DX-S^®^ is close to two times higher than the DQE of a PIP based CR system in the spatial frequency range from 0 to 5 lp/mm (Figure 36), which implies that, theoretically, similar image quality is obtained at only half the X-ray dose.

A thorough analysis was done of the improvement in technical image quality of DX-S^®^ with NIP with respect to a PIP based CR system [111]. The conclusion was that the higher DQE of DX-S^®^ is not only due to a higher quantum efficiency and MTF, but also to lower screen-structure noise and lower secondary quantum and Swank noise.

The prediction based on technical image quality evaluations, that a strong reduction in X-ray dose would be possible, was confirmed in several clinical or detail visibility studies:

 (1) The Institute for Clinical Radiology in Munich evaluated image quality and anatomical detail depiction [112]. Dose-reduced radiographs were made with NIP and compared to full dose PIP images. 24 Supine chest radiographs were made with PIP at standard dose and compared to follow-up studies with NIP in which the dose was reduced by 50%. In a blind study, 6 independent readers rated the PIP and dose-reduced NIP images equally. The same group also evaluated the low-contrast performance of DX-S^®^ compared to that of a PIP CR system [113]. A total of 36 images of a CDRAD phantom were made using nine different exposure conditions. A blind study of the images by five radiologists and five physicists lead to the conclusion that for all but two of the exposure settings NIP allowed visualization of significantly lower contrast levels. The remaining two settings also showed a trend toward better low contrast depiction with NIP.
 (2) A comparison of imaging performance with different doses in skeletal radiography was made for a PIP based CR system, DX-S^®^ and a DR system [114]. The DR system was a Siemens flat-panel detector based on a CsI:Tl^+^ scintillator layer and an amorphous silicon sensor layer. Five independent blinded radiologists evaluated 72 plain radiographs of the feet of six human cadavers obtained with four surface entrance doses. The conclusion was that the radiation dose can be reduced by 75% in clinical skeletal imaging of peripheral extremities when NIP is used instead of PIP in CR. The DR system allowed a dose reduction of 50%.
 (3) Physical image quality was compared for two CR and two flat-panel DR systems [115]. The CR systems were DX-S^®^ and the PIP based Agfa ADC Compact Plus^®^. The first DR system, DR1, was a General Electric Revolution XQ/I^®^ with an a-Si panel and a CsI:Tl^+^ scintillator; the second, DR2, was a Philips Diagnost^®^, based on the same technology. Image quality was assessed with a contrast-detail object and acrylic material to simulate clinical conditions. Important image quality differences were observed. DX-S^®^ and DR2 showed similar image quality and were superior compared to DR1 and ADC Compact Plus^®^. It was further concluded that DX-S^®^ provides better low-contrast detectability and a potential for dose reduction and that for doses over 0.2 mGy it provides even better image quality than DR1 and DR2.
 (4) Smans *et al*. tested CR image quality in neonatal imaging [116]. For typical acquisition parameters of neonatal chest X-ray examinations, the threshold-contrast detectability in simulated and acquired images of a contrast-detail phantom was compared. This was done for the Agfa ADC Compact Plus^®^ with PIP and for DX-S^®^. Good agreement was found between the threshold-contrast curves of the simulated and experimentally acquired images and the superiority of NIP for neonatal imaging was confirmed.
 (5) Ludewig *et al*. simulated the conditions in neonatal radiological practice by making thoracic radiographs of cats. They compared DX-S^®^ to ADC Compact Plus^®^. The conclusion was that a dose reduction of 50% seems possible without relevant deterioration of image quality [117].

The benefit of improved light collection in a line-scanning system had already been demonstrated by Herrmann *et al*. [118]. The DQE at high spatial frequencies improves significantly with increasing system gain. This improved line scanner was never commercialized.

**Figure 37:**
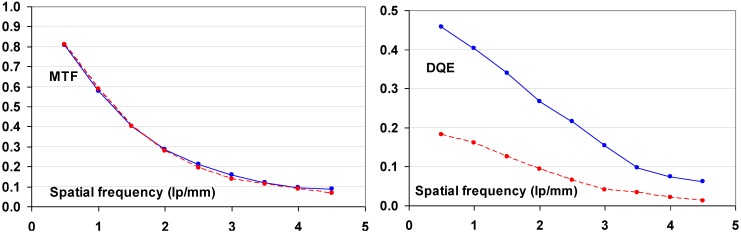
Average MTF and DQE for RQA 5 beam quality for PIP and NIP in DX-G^®^. MTF is measured at ~8 μGy, DQE is given for 2.5 μGy dose.

**Figure 38:**
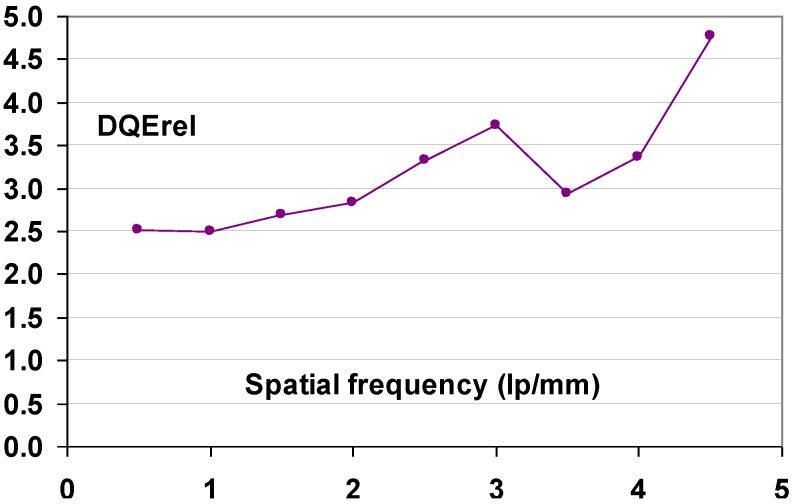
Ratio of NIP DQE to PIP DQE in DX-G^®^. RQA 5 exposure and 2.5 μGy dose.

In 2010 Agfa introduced DX-G^®^, a flying spot CR system for general radiography capable of reading both PIP and NIP. Improved image quality is reached for NIP in DX-G^®^ thanks to the higher light collection efficiency and, therefore, higher gain, G, as compared to the DX-S^®^ system. In Figure 37 the MTF of PIP and NIP are compared in DX-G^®^ for RQA 5 beam quality. In the current design of image plates, with about a two times thicker phosphor layer for NIP, the MTF is similar for the two plates. As a result of the higher absorption and the higher conversion, the DQE for NIP is between 2.5 and 5 times higher than for PIP in the same digitizer (Figure 38).

**Figure 39:**
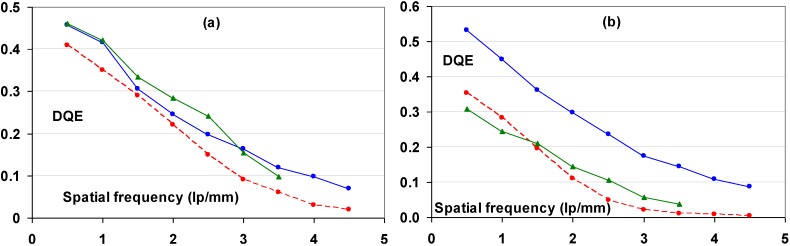
DQE of DX-S^®^ with NIP (--•--) of DX-G^®^ with NIP (−•−) and of DX-D 500^®^ (−▲−) for 2.5 μGy exposure under RQA 5 (**a**) and RQA 3 (**b**) exposure conditions.

The current status of image quality with NIP in DX-G^®^ is by far the best ever reached for CR. In Figure 39 the DQE for DX-G^®^ and DX-S^®^ with NIP is compared. To illustrate that the current status of CR reaches a high image quality level, the DQE for the Agfa DR system DX-D 500^®^ (based on CsI needle scintillator plate), is also included. The DQE of DX-G^®^ with NIP is similar to that offered by the DR system. It is about 20% higher at lower spatial frequencies and up to more than two times higher at high spatial frequencies than the DQE of the same plate in the DX-S^®^ line scanner. Thanks to the fact that DX-G^®^ does not suffer from electronic noise and that for NIP screen structure noise is also very limited, the DQE for the DX-G^®^ + NIP system is quasi dose independent. This makes the DX-G^®^ especially fit for low dose applications. DR systems have a high contribution of electronic noise which deteriorates the DQE at low dose. At low dose, the DX-G^®^ + NIP system prevails even over DR systems. 

For RQA 3 beam quality, which typically represents extremity applications and the more critical paediatric applications, the DQE of DX-G^®^ with NIP significantly exceeds that of the best DR systems (Figure 39b). Advantageous for the DX-G^®^ + NIP system are the higher X-ray absorption, higher system gain and no electronic noise contributions. DR systems have an easier workflow in the hospital and are known for their good image quality and are therefore strongly appreciated. Although the workflow for CR is somewhat more demanding for the operators, the better image quality or lower patient dose for the same image quality in some applications makes the new generation of CR systems highly competitive and to some extend complementary to DR systems.

**Figure 40:**
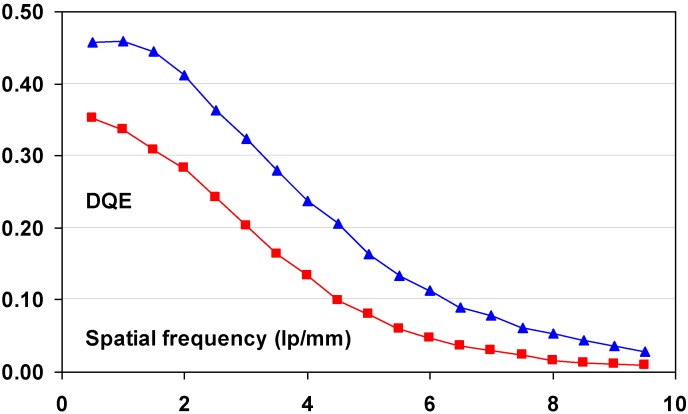
Measured PIP (red squares) and NIP (blue triangles) CR systems’ DQE (lines) (data from [80]).

In 2010 Agfa introduced DX-M^®^, a CR system suitable for general radiography and mammography, based on a flying-spot scanner and capable of reading both PIP and NIP. For mammography, technical image quality parameters as MTF and DQE, were measured for both plate types and simulated using a linear system approach [80]. DQE was 30% higher for NIP at low spatial frequencies and 50% higher at 5 lp/mm (Figure 40). The MTF is only slightly higher and contributes only marginally to the better image quality. Since with the low-energy mammography spectrum, X-ray absorption is high for both PIP and NIP, there is not an important difference in X-ray quantum noise. The benefit of NIP for mammo results from the higher transparency of the phosphor layer, allows higher read-out depths and PSL escape efficiencies and, therefore, a higher system gain. The lower degree of screen-structure noise also contributes. The expectation is that the mammo NIP will make a reduction in X-ray dose of 30 to 50% possible.

An investigation by Peloschek *et al*. [119] leads to the conclusion that the needle-based CR system (DX-M^®^ digitizer with CR HM 5.0^®^ NIP detector) operates at a dose that is 40% higher than that of existing DR systems (GE Essential^®^, Hologic-Dimensions^®^, Siemens-Inspiration^®^). However, in comparison to CR systems with PIP the dose is 25 to 40% lower; and the system shows a significant dose reduction for the screening-relevant population (50 years and older) compared to the previously used screen-film system (Kodak MIN-R^®^).

## 10. Outlook

CR and DR are competing technologies that already coexist for the past 15 years. Both have their stronger and weaker points. Image quality of both systems depends heavily on the type of phosphor used. As has been demonstrated here, CR with NIP can match DR with CsI:Tl scintillator. CR with PIP offers similar image quality as DR with Gd_2_O_2_S:Tb powder scintillators. DR allows higher productivity, but CR has the advantage of portability and flexibility, particularly in the OR and trauma areas and for neonatal imaging. Until now, DR has been more costly than CR, but its cost is coming down. More compact and less costly CR systems are being developed, to conquer the low end market. For these reasons, it is believed that the two imaging technologies will still coexist for a long time.

As a result, phosphors will keep playing an important role in medical X-ray imaging, as storage medium in CR and as scintillator in DR. The authors are not aware of new phosphors that are in development for any of the two technologies. Materials with a higher density than the Cs halides and that allow deposition of needle layers would, in principle, allow a next quantum step in image quality. Gradual further improvement of the needle layers is expected by technologies that further suppress lateral light scatter.

An alternative DR technology is based on direct conversion detectors. A semiconductor material converts X-ray quanta directly in electron/hole pairs, without the need of a phosphor creating the visible light intermediate. a-Se has proven to be an adequate material for a direct conversion mammography detector. If a similarly suitable material could be developed for general radiography, further image quality improvement could be realized. Photoconductors with high X-ray absorption across the general radiography energy spectrum are under investigation. Examples are polycrystalline materials as lead iodide, mercury iodide, lead oxide, and cadmium-zinc-telluride.

## 11. Conclutions

A prerequisite for a medical CR storage phosphor is high X-ray absorption. In the interest of the patient medical doses are kept low. Hence, X-ray quantum noise is the most important noise source in images from decent CR systems and the CR plate should use the X-ray quanta as economically as possible. That is, it should have an absorption that is as high as possible. It is not surprising that the commercial CR phosphors today are phosphors of the BaFX:Eu^2+^ family and CsBr:Eu^2+^. Both materials have a relatively high density of about 5 g/cm^3^ and contain an element with a K-edge between 35 and 40 keV, matching well with the X-ray spectra used in general radiography.

Most CR scanners are flying-spot scanners with a PMT light detector. Since amplification noise is a second important noise source, also the photons emitted by the phosphor should be used as economically as possible. The PMT conversion efficiency being higher for higher photon energies, the UV to blue Eu^2+^ emission is very suitable for reaching a high gain in CR. In addition, the Eu^2+^ luminescence fulfils the requirement of having a decay-time short enough for efficient read-out in a flying-spot scanner. In hindsight, it is no surprise that both important CR phosphor classes have Eu^2+^ as activator.

Suitable defects are required for PSL. F centers, based on vacancies of monovalent ions, are good candidates to act as electron traps from which trapped electrons can be stimulated with relatively low energy. A lower valence of the missing ion corresponds to a lower binding energy of the electron in the vacancy and to longer stimulation wavelengths. The larger monovalent ions lead to larger F centers and are, therefore, the preferred F center sources. This explains why red-stimulable F centers are created in Br^−^ containing compounds as BaFBr:Eu^2+^ and CsBr:Eu^2+^. I^−^ is even more preferred and partial or complete replacement of Br^−^ in BaFBr:Eu^2+^ has lead to a further improvement of the storage phosphor. For some reason, CsI seems to be no suitable host material for a room-temperature storage phosphor. Further defects or impurities are required for PSL that can act as hole trap and, as it turns out, as stabilizing agents. O^2−^ is incorporated in BaFBr:Eu^2+^ on a halide position and plays the role of hole trap. In (Ba,Sr)F_1+x_Br_1−x_:Eu^2+^ this role is taken over by F^−^ antisites, *i.e*., F^−^ ions on a Br^−^ lattice positions. It is the only medical CR phosphor that does not rely on oxygen or water as essential impurity. There is strong evidence that H_2_O plays an eminent role in CsBr:Eu^2+^ by stabilizing the Eu^2+^ monomers, and preventing their agglomeration. Agglomeration would make the material inactive as storage phosphor. Since the hole trap has not been identified yet in CsBr:Eu^2+^, it is not impossible that, as in BaFBr:Eu^2+^, O^2−^ serves as hole trap. It seems likely that a slightly hygroscopic character of the mother lattice is a favorable material property to make a storage phosphor. It will help incorporating the abovementioned impurities when the phosphor is produced in a solid state process.

As in image intensifiers and DR, the morphology of the phosphor crystals in the CR imaging plate has a very significant impact on its performance. The light-guiding character of the crystals in NIP leads to a large benefit over PIP. It allows the use of much thicker phosphor layers in general radiography that have at least a two times higher X-ray absorption. The higher light transparency further leads to a higher system gain and the IP’s are more homogeneous and lead to less screen-structure noise. It has been demonstrated that, for many general radiography applications, NIP leads to the same image quality as PIP at 50% or less of the X-ray dose. CR with NIP offers the same image quality for most general radiography applications as DR with a CsI:Tl^+^ needle scintillator plate. When small detail visibility is important and when a softer X-ray beam is used as in paediatry and imaging of extremities, it even leads to significantly better image quality than this competing technology. DQE comparisons and first clinical evaluations indicate that in mammography a reduction in X-ray dose of 30 to 50% is possible with NIP.
